# Topology and function of translocated EspZ

**DOI:** 10.1128/mbio.00752-23

**Published:** 2023-06-21

**Authors:** Nir Haritan, Etan Amse Bouman, Ipsita Nandi, Raisa Shtuhin-Rahav, Efrat Zlotkin-Rivkin, Tsafi Danieli, Naomi Melamed-Book, Yael Nir-Keren, Benjamin Aroeti

**Affiliations:** 1 Department of Biological Chemistry, Alexander Silberman Institute of Life Sciences, The Hebrew University of Jerusalem, Jerusalem, Israel; 2 The Protein Production Facility, Wolfson Centre for Applied Structural Biology, Alexander Silberman Institute of Life Sciences, The Hebrew University of Jerusalem, Jerusalem, Israel; 3 Bioimaging Unit, Alexander Silberman Institute of Life Sciences, The Hebrew University of Jerusalem, Jerusalem, Israel; University of Washington, Seattle, Washington, USA

**Keywords:** enteropathogenic *E. coli*, type III secreted effectors, EspZ, Tir, cell death, mitochondria, bacterial colonization, host–pathogen interactions

## Abstract

EspZ and Tir are essential virulence effectors of enteropathogenic *Escherichia coli* (EPEC). EspZ, the second translocated effector, has been suggested to antagonize host cell death induced by the first translocated effector, Tir (translocated intimin receptor). Another characteristic of EspZ is its localization to host mitochondria. However, studies that explored the mitochondrial localization of EspZ have examined the ectopically expressed effector and not the more physiologically relevant translocated effector. Here, we confirmed the membrane topology of translocated EspZ at infection sites and the involvement of Tir in confining its localization to these sites. Unlike the ectopically expressed EspZ, the translocated EspZ did not colocalize with mitochondrial markers. Moreover, no correlation has been found between the capacity of ectopically expressed EspZ to target mitochondria and the ability of translocated EspZ to protect against cell death. Translocated EspZ may have to some extent diminished F-actin pedestal formation induced by Tir but has a marked effect on protecting against host cell death and on promoting host colonization by the bacteria. Taken together, our results suggest that EspZ plays an essential role in facilitating bacterial colonization, likely by antagonizing cell death mediated by Tir at the onset of bacterial infection. This activity of EspZ, which occurs by targeting host membrane components at infection sites, and not mitochondria, may contribute to successful bacterial colonization of the infected intestine.

**IMPORTANCE:**

EPEC is an important human pathogen that causes acute infantile diarrhea. EspZ is an essential virulence effector protein translocated from the bacterium into the host cells. Detailed knowledge of its mechanisms of action is, therefore, critical for better understanding the EPEC disease. We show that Tir, the first translocated effector, confines the localization of EspZ, the second translocated effector, to infection sites. This activity is important for antagonizing the pro-cell death activity conferred by Tir. Moreover, we show that translocated EspZ leads to effective bacterial colonization of the host. Hence, our data suggest that translocated EspZ is essential because it confers host cell survival to allow bacterial colonization at an early stage of bacterial infection. It performs these activities by targeting host membrane components at infection sites. Identifying these targets is critical for elucidating the molecular mechanism underlying the EspZ activity and the EPEC disease.

## INTRODUCTION

Enteropathogenic and enterohemorrhagic *Escherichia coli* (EPEC and EHEC, respectively) are attaching and effacing (A/E) enteric bacterial diarrheal pathogens causing significant morbidity and mortality worldwide (1 - 3). A/E pathogens infect the apical surface of the intestine’s enterocytes, where they locally efface their microvilli and attach firmly to the cell surface. The type III secretion system (T3SS) encoded on a pathogenicity island called the locus of enterocyte effacement plays an important role in exerting the A/E effect. The effect is partly contributed by the T3SS-dependent translocation (injection) of bacterial proteins, called “effectors,” from the bacterium into the infected enterocytes (4 - 6).

Intimate bacterial attachment to the cells is mediated by the first translocated and the most abundant effector, the translocated intimin receptor (Tir). Tir is inserted into the host plasma membrane and interacts via its extracellular loop with the bacterial outer membrane adhesin protein, intimin (7). Tir–intimin interactions trigger marked rearrangements of the actin cytoskeleton, resulting in the assembly of the actin cytoskeleton and the formation of filamentous (F) actin-rich membrane protrusions called “pedestals” on top of which the bacterium resides (8). After Tir, additional effectors are injected (translocated) in a particular spatiotemporal order (9, 10).

EspZ (SepZ) is the second translocated and the second most abundant effector (9, 10). Tir and EspZ are the only known EPEC effectors suggested to adopt a hairpin-like conformation containing two membrane-spanning domains and *N*- and *C*-termini facing the host cell cytoplasm (7, 11). Studies on infected mice (12) and rabbits (13) have shown that the effector is essential for exerting the EPEC disease. Once translocated into the host cell, EspZ is detected beneath the sites of bacterial attachment nearby translocated Tir and pedestals (14). Remarkably, EspZ has been suggested to exert inhibition of translocation of subsequent effectors (11, 13, 15), a hypothesis that could be supported by the observation that the effector interacts with the EPEC type III translocon protein, EspD (16).

A prominent hallmark of EspZ is the capacity to inhibit epithelial cell death (13, 17, 18). This has been suggested to occur by several mechanisms, including the ability to block the type III secretion of pro-death effectors (e.g., EspF and Map), the capability to interact with host pro-survival protein CD98 (19), and the capability to interact and alter the morphological and functional properties of host mitochondria (17, 20). However, these studies tested the ectopically expressed effector, which could be physiologically less relevant than the translocated one.

Recent studies using EPEC-expressing Tir only (termed EPEC1) or EPEC expressing Tir and EspZ only (termed EPEC2) have shown that clustering of Tir during EPEC infection triggers calcium influx-dependent inflammatory cell death in intestinal epithelial cells. Intriguingly, the subsequently translocated EspZ contributed by cell infection with EPEC2 negated the Tir-triggered cell death (21, 22). This suggests that inflammatory cell death is exerted at the onset of EPEC infection by Tir and inhibited immediately thereafter by translocated EspZ.

Here we show that translocated EspZ localizes at infection foci consisting of an extracellular loop linking the two predicted transmembrane domains, and of *C*- (and likely the *N-*) termini projecting into the host cell cytoplasm. Translocated EspZ did not localize to host mitochondria, yet confers protection against host cell death. Moreover, we show that translocated Tir is important for determining the localization of EspZ at infection sites, where it protects against Tir-induced host cell death.

## RESULTS

### Translocated EspZ-2xHA-SBP is localized to infection sites and protects against EPEC-ΔespZ* (an EPEC strain mutated in espZ and effector genes encoded by the pp4/IE6 islands)–mediated cell death

**Fig 1:**
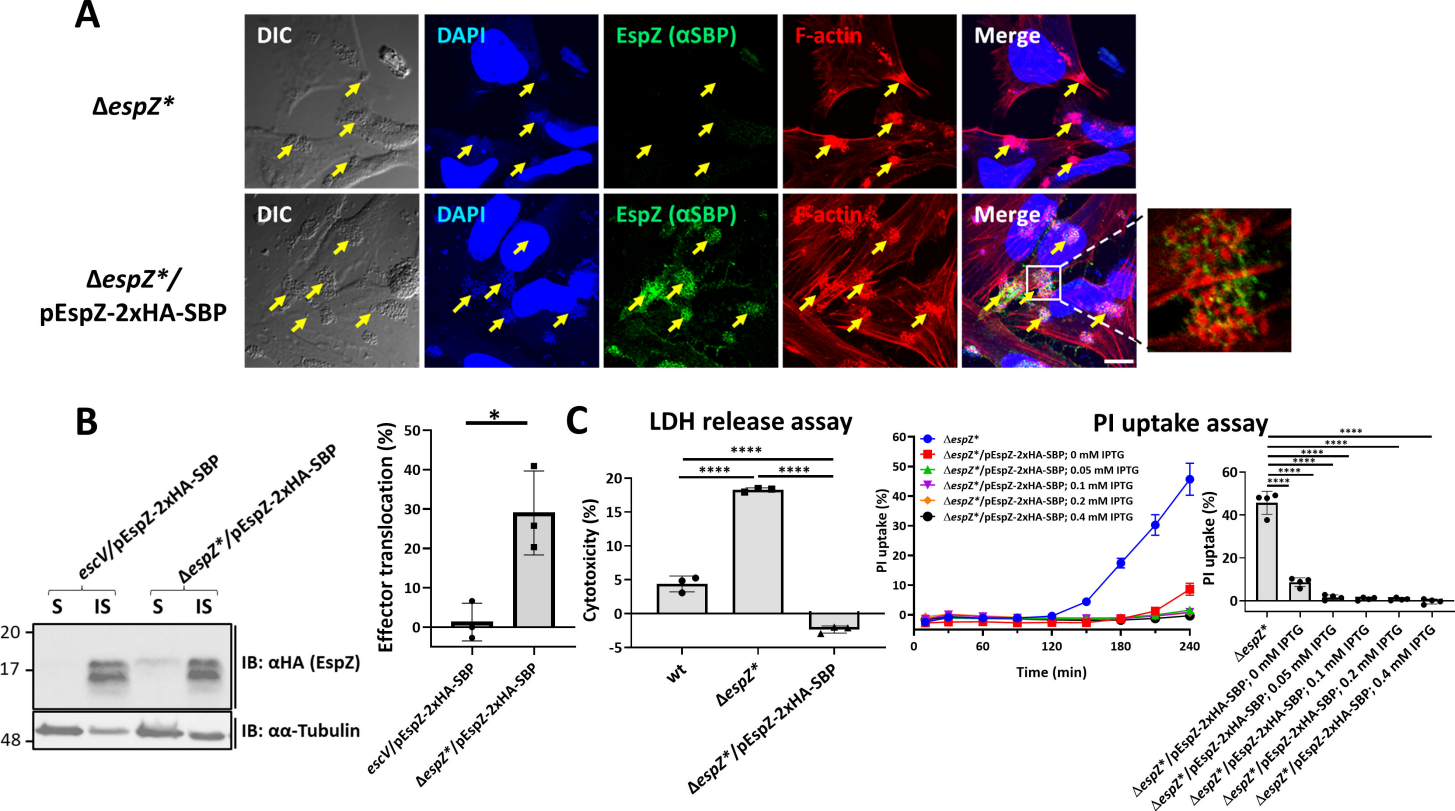
Translocated EspZ is localized at infection sites and confers protection against host cell death induced by EPEC-ΔespZ*. (**A**) Confocal imaging. HeLa cells were infected with pre-activated EPEC-ΔespZ* or EPEC-ΔespZ*/pEspZ-2xHA-SBP (schematically illustrated in Fig. S1) for 60 minutes at 37°C. The cells were then fixed, permeabilized, and labeled with anti-SBP antibodies followed by Alexa Fluor 488 anti-mouse secondary antibodies (to visualize EspZ). The cells were then stained with TR-phalloidin (to visualize F-actin) and DAPI (to visualize host nuclei and bacterial DNA), fixed, and analyzed by confocal microscopy. Representative images (out of three independent experiments) are shown. Inset is used to show a small portion of the image at higher magnification. Arrows point toward bacterial infection sites. Scale bar, 10 µm. (**B**) EspZ translocation. The “effector translocation assay” was applied to evaluate the translocation level of EspZ into the host cells, as described in the Materials and Methods section. A representative (out of three independent experiments) immunoblot (IB, left) and the effector translocation levels in percentage (right) are shown. Results are mean ± SD. *P-value ≤ 0.05. (**C**) Effects on lytic cell death. HeLa cells were infected with the indicated EPEC strains, and the lactate dehydrogenase (LDH) release or propidium iodide (PI) uptake assays were performed to estimate the effects of bacterial infection on the host (lytic) cell death (see Materials and Methods). Graphs of cell cytotoxicity levels determined by the LDH release assay (left), and PI uptake levels as a function of infection time, and the last time of infection (*T* = 240 minutes) (right) are shown. Results are mean ± SD. **** *P*-value < 0.0001.

An EspZ-2xHA-SBP (influenza hemagglutinin-tag (HA)-streptavidin-binding peptide tag (SBP)) (schematically shown in Fig. S1) encoding construct was made in a pSA10 expression vector. The plasmid was electroporated into EPEC-ΔespZ* to generate the EPEC-ΔespZ*/pEspZ-2xHA-SBP-complemented bacterial strain. HeLa cells infected with EPEC-ΔespZ* or EPEC-ΔespZ*/pEspZ-2xHA-SBP were immunostained with anti-SBP antibodies to visualize EspZ and with 4′,6-diamidino-2-phenylindole (DAPI) and TR-phalloidin that labeled bacterial colonies and F-actin rich pedestals, respectively. Confocal imaging shows that in the EPEC-ΔespZ*/pEspZ-2xHA-SBP-infected cells, EspZ is located juxtaposed to bacterial attachment sites and F-actin foci. No immunostaining was observed in EPEC-ΔespZ*-infected cells, indicating that the EspZ labeling is specific (Fig. 1A). Using the effector translocation assay, we demonstrate that EspZ-2xHA-SBP is translocated into cells infected with EPEC-ΔespZ*/pEspZ-2xHA-SBP but not into cells infected with EPEC-*escV*/pEspZ-2xHA-SBP (an EPEC strain with a null *escV* gene of the T3SS and is therefore unable to assemble the T3SS and to translocate effectors) (Fig. 1B), suggesting that the effector protein is translocated into the cells in a T3SS-dependent manner.

The mechanism by which EPEC effectors induce regulated cell death is complex. It involves intrinsic and extrinsic pathways of apoptosis, necroptosis, and pyroptosis (21 - 26). While the three pathways have a profound effect on the physical properties of the host cell plasma membrane, only the latter two trigger plasma membrane perforation and lytic cell death (27). EspZ has been suggested to inhibit the apoptotic, necrotic (18), and lytic pyroptotic (22) cell death routes. We, as others (18, 21), investigated the EspZ-mediated regulation of host cell death using the lactate dehydrogenase (LDH) and propidium iodide (PI) uptake assays, which report for the lytic pathways. Using the LDH release assay, we show that infection with EPEC-ΔespZ* caused greater host cell cytotoxicity compared to infection with EPEC-*wt*. In contrast, no-host cytotoxicity was observed on infection with EPEC-ΔespZ*/pEspZ-2xHA-SBP (Fig. 1C). The PI uptake assay showed a marked increase in PI uptake levels as a function of infection time was observed in cells infected with EPEC-ΔespZ*. Conversely, a minimal increase in PI uptake was observed only after ~200 minutes of infection in cells infected with EPEC-ΔespZ*/pEspZ-2xHA-SBP that were not treated with IPTG (0 mM IPTG) (Fig. 1C). This could be attributed to minimal induction of EspZ that is not enough to induce robust protection against EPEC-Δ*espZ*-mediated host cell death. PI uptake remained at nearly zero levels in cells infected with EPEC-ΔespZ*/pEspZ-2xHA-SBP whose EspZ expression was induced with 0.05 mM or higher IPTG concentrations (Fig. 1C). These results combined, which are consistent with previous reports (13, 14, 18), suggest that the translocated effector effectively protects against EPEC-ΔespZ*-induced host cell death. Similar results were obtained with an EspZ-2xHA-encoding construct (Fig. S2), suggesting that the *C*-terminal SBP tag does not alter the functionality of EspZ-2xHA. Additionally, the two tagged versions of EspZ mimic faithfully the activity of genomic- (18) or plasmid- (11) encoded non-tagged versions of EspZ, concerning the protection against host cell death activities.

### Topology of translocated EspZ

Previous studies examining the short extracellular loop of EPEC and EHEC EspZ suggested that the loop is involved in determining the strain-specific induction of pedestal formation and cell death activities (11). However, these studies did not provide direct evidence for the topology of the loop. To investigate this, the FLAG-tag and Tobacco Etch Virus Protease (TEV) recognition site were introduced into the putative extracellular loop of EspZ-2xHA-SBP (schematically shown in Fig. S3A) to generate an EspZ-FLAG-TEV-2xHA-SBP encoding pSA10 constructs. The vector was electroporated into EPEC-ΔespZ*, or into the EPEC-*escV*, to produce the EPEC-ΔespZ*/pEspZ-FLAG-TEV-2xHA-SBP and EPEC-*escV*/pEspZ-FLAG-TEV-2xHA-SBP complemented strains, respectively. Using the effector translocation assay, we could demonstrate that EspZ-FLAG-TEV-2xHA-SBP is translocated into infected HeLa cells in a T3SS-dependent fashion (Fig. S3B). It also confers protection against cell death induced by EPEC-ΔespZ* infection (Fig. 4B), suggesting that the loop-introduced tags do not interfere with the anti-cell death function of EspZ.

**Fig 2:**
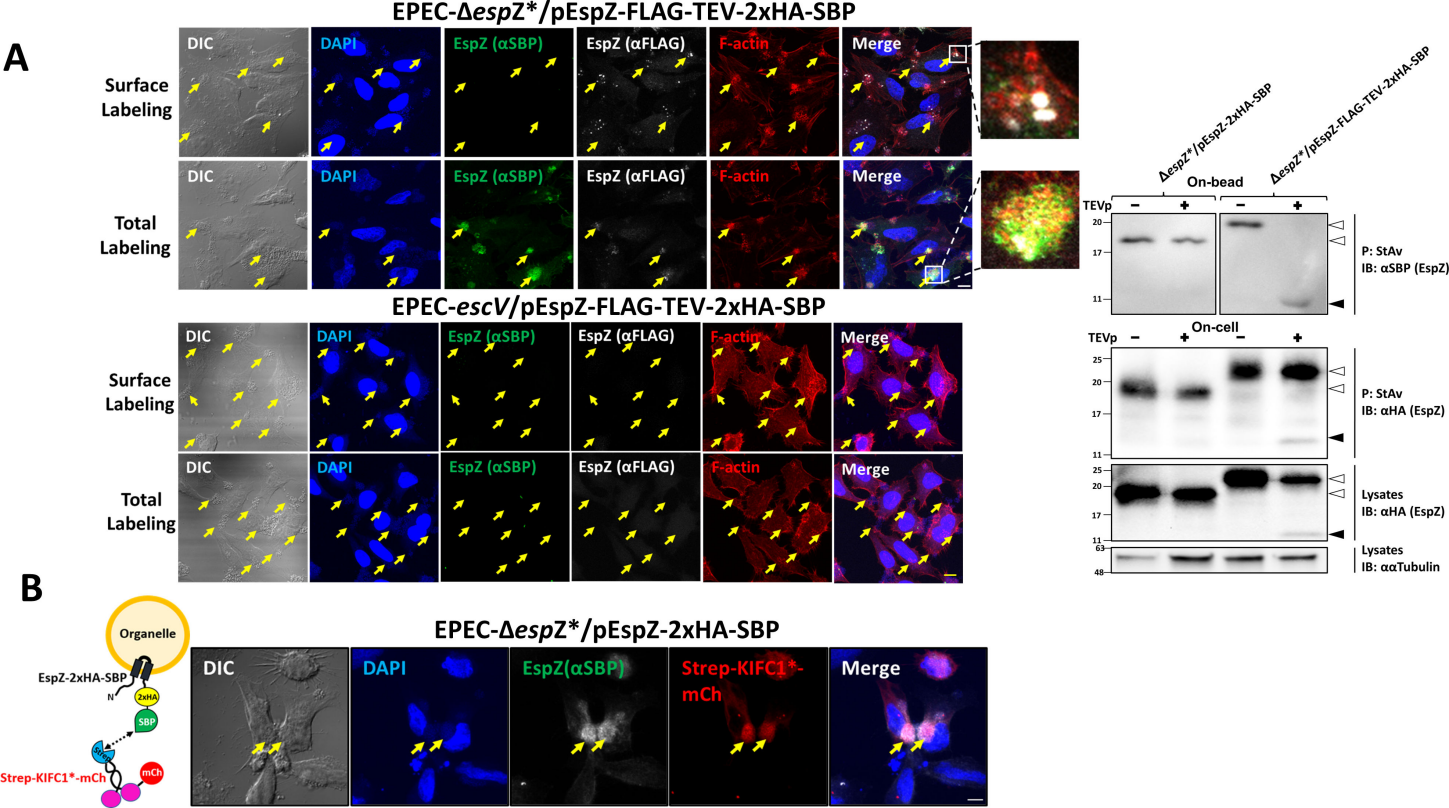
Membrane topology of translocated EspZ. (**A**) The loop of EspZ is exposed to the extracellular environment. Analysis by surface labeling (left): HeLa cells were infected with EPEC-ΔespZ*/pEspZ-FLAG-TEV-2xHA-SBP or EPEC-*escV*/pEspZ-FLAG-TEV-2xHA-SBP for 45 minutes at 37°C. Cells were then subjected to “Surface” or “Total” immunolabeling, as described in Materials and Methods section. Representative images out of three independent experiments are shown. Arrows point toward infecting bacterial microcolonies. Insets are used to show a small portion of the image at higher magnification. Bar, 10 µm. Analysis by TEVp cleavage (right): HeLa cells were infected with EPEC-ΔespZ*/pEspZ-2xHA-SBP or with EPEC-ΔespZ*/pEspZ-FLAG-TEV-2xHA-SBP. EspZ pulled down with streptavidin agarose was subjected to TEVp using the “On-bead” (upper) or “On-cell” (lower) digestion protocols, as described in Materials and Methods section. The presence of precipitated EspZ (P) and EspZ in cell lysates (Lysates) were analyzed by IB. The intact (white arrowheads) and cleaved (black arrowheads) effector were identified by IB. Representative (out of three independent experiments) immunoblots from three independent experiments are shown. (B) The C-terminus of translocated EspZ-2xHA-SBP contacts the host cell cytoplasm*.* A plasmid encoding the cytoplasmic microtubule minus-end streptavidin (strep)-tagged KIF1C fused to mCherry (Strep-KIFC1*-mCh [28], illustrated in the diagram) was ectopically expressed for 24 hours in HeLa cells. Cells were subsequently infected (45 minutes at 37°C) with EPEC-ΔespZ*/pEspZ-2xHA-SBP, fixed, permeabilized, and immunostained with anti-SBP followed by Alexa-Fluor 488 anti-mouse secondary antibodies to visualize EspZ and then stained with DAPI (to visualize host nuclei and bacterial DNA). Cells were further processed for confocal imaging, as described in Materials and Methods section. The streptavidin (Strep) in the expressed cytoplasmic motor is expected to interact with the *C*-terminal SBP of the translocated effector (illustrated as a dashed double arrowhead line in the diagram), resulting in colocalization between the two proteins. Arrows point toward infecting bacterial microcolonies where significant colocalization between the Strep-KIFC1*-mCh and translocated EspZ is observed. Representative images (from three independent experiments) are shown. Bar, 10 µm.

To test whether the loop linking the two transmembrane domains faces the extracellular environment, HeLa cells were infected with EPEC-ΔespZ*/pEspZ-FLAG-TEV-2xHA-SBP or EPEC-*escV*/pEspZ-FLAG-TEV-2xHA-SBP. The infected cells were then exposed concomitantly to rabbit anti-FLAG and mouse anti-SBP antibodies in the cold (4°C). Under these conditions, the anti-FLAG antibodies are expected to bind the FLAG epitope if the loop is exposed to the extracellular environment (Surface Labeling). In contrast, the anti-SBP antibodies will not bind SBP if the *C*-terminus projects into the host cell cytoplasm. Cells were labeled with the appropriate fluorescently tagged secondary antibodies, fixed, permeabilized, and stained with DAPI (DNA, host nuclei and bacteria) and TR-phalloidin (F-actin, pedestals). In another experiment, cells were infected in the same way, except that the FLAG and SBP tags were immunostained with the antibodies after cell fixation and permeabilization. Under these conditions, the entire EspZ population is expected to be visualized in the infected cells (Total Labeling). Identical experiments were performed in EPEC-*escV*/pEspZ-FLAG-TEV-2x-HA-SBP-infected cells, which are not expected to inject the effector protein and not stained with either antibody. Confocal imaging of EPEC-ΔespZ*/pEspZ-FLAG-TEV-2xHA-SBP-infected cells indeed showed a clear punctate fluorescence labeling of the anti-FLAG antibodies close to infection sites but not with the anti-SBP antibodies (Fig. 2A, upper left; “Surface Labeling”). Conversely, apparent fluorescence staining of anti-FLAG and anti-SBP antibodies was observed in the permeabilized cells (Fig. 2A, upper left; “Total Labeling”). As expected, confocal imaging of EPEC-*escV*/pEspZ-FLAG-TEV-2xHA-SBP-infected cells shows neither surface nor total fluorescence labeling using these antibodies (Fig. 2A, lower left).

**Fig 3:**
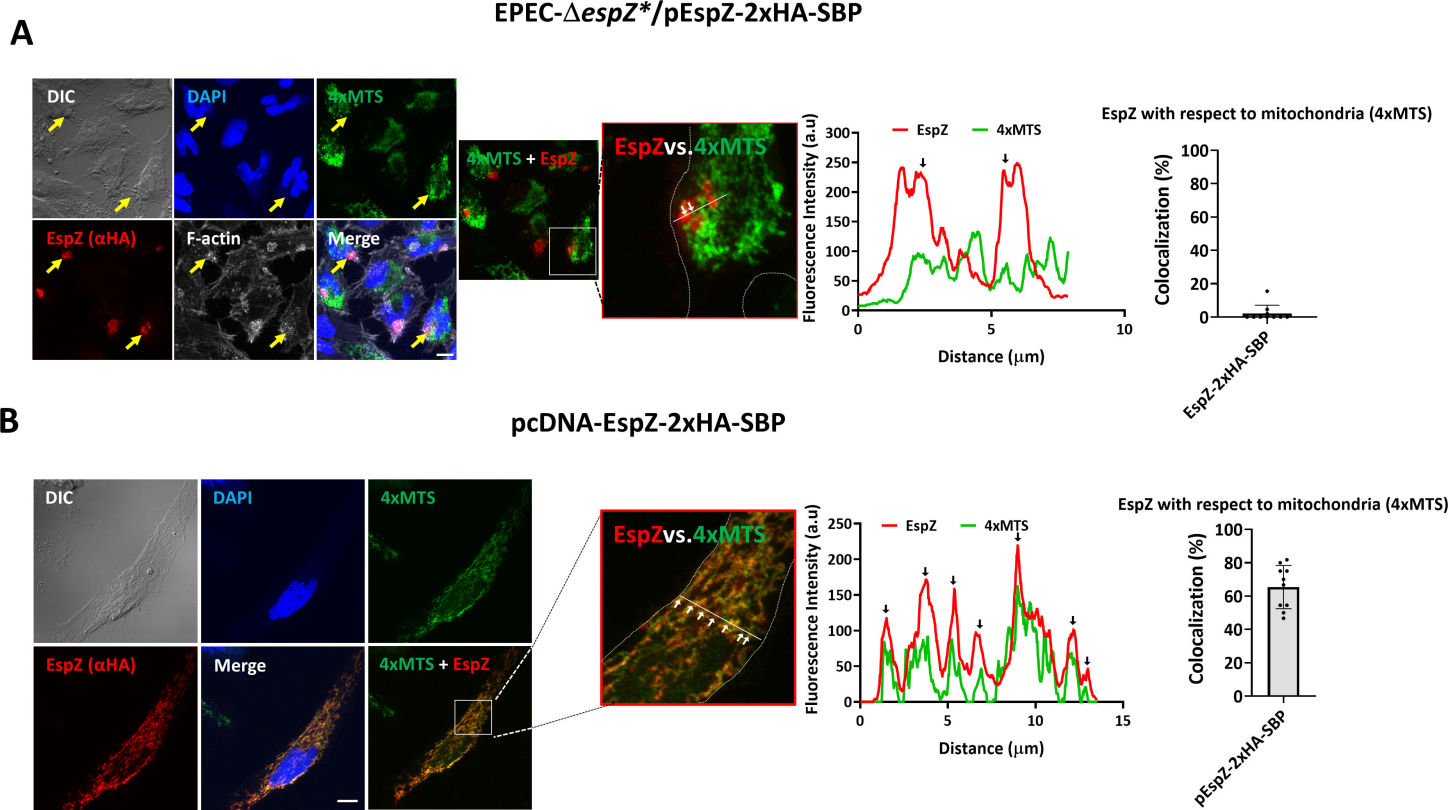
A minor fraction of translocated EspZ and a significant fraction of the ectopically expressed effector localizes to mitochondria, respectively. (**A**) Translocated EspZ localization to mitochondria. HeLa cells ectopically expressing the mitochondrial marker 4xMTS-mNeonGreen were infected with EPEC-ΔespZ*/pEspZ-2xHA-SBP for 45 minutes at 37°C. Cells were fixed, permeabilized, and immunostained with anti-HA antibodies and appropriate fluorescently labeled secondary antibodies, to visualize EspZ. Cells were also stained with DAPI (DNA) and Phalloidin CF 647 (F-actin). Cells were imaged by confocal microscopy and representative images (out of three independent experiments) are shown. Yellow arrows point toward infecting bacteria. An enlargement of the boxed region with a line used to generate a fluorescence intensity profile of the EspZ (red) and 4xMTS (green) fluorescence is shown in the red boxed image as a representative example. Cell edges are indicated with a dashed white line in that image. While arrows pointing toward copeaking fluorescence intensity signals are shown in the magnified image and the line graph. The degree (%) of EspZ versus 4xMTS colocalization is shown in a bar chart. Results are mean ± SD. Bar, 10 µm. (**B**) Ectopically expressed EspZ localization to mitochondria. HeLa cells were cotransfected with pcDNA encoding 4xMTS-mNeonGreen and EspZ-2xHA-SBP. After 24 hours, cells were fixed, immunostained with anti-HA (to label EspZ), stained with DAPI (DNA), and processed for confocal microscopy. Out of three independent experiments, representative images are shown in the left panel. Bar, 10 µm. The boxed region is enlarged, in which the cell edges are indicated with a white dashed line is shown. Arrows in the image and the fluorescence intensity graph point toward colocalizing 4xMTS and EspZ signals. The degree (%) of colocalization is shown in a bar chart. The results are the mean ± SD.

**Fig 4:**
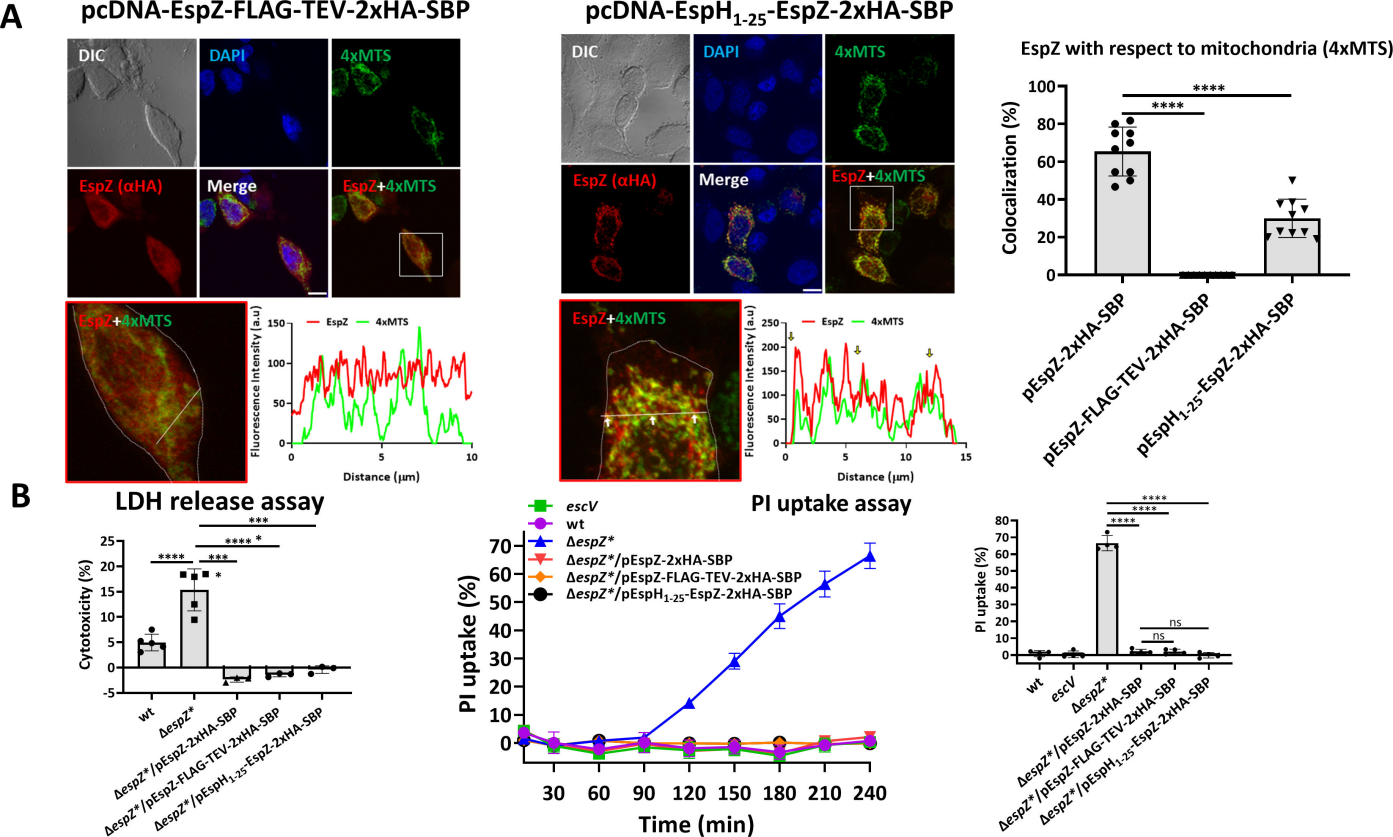
Ectopically expressed EspZ localizes to mitochondria in a manner that does not correlate with the capacity of the translocated effector to protect against host cell death. (**A**) Colocalization analysis of ectopically expressed EspZ vis-à-vis mitochondria. EspZ-FLAG-TEV-2xHA-SBP or EspH_1-25_-EspZ-2xHA-SBP (illustrated in Fig. S3A and Fig. S5A, respectively) encoding pcDNA (mammalian expression vector) plasmids (pcDNA-EspZ-FLAG-TEV-2xHA-SBP and pcDNA-EspH_1-25_-EspZ-2xHA-SBP) were coexpressed with 4xMTS-mNeonGreen in HeLa cells. Cells were fixed, immunostained with anti-HA antibodies (to visualize EspZ), and then stained with DAPI (to image cell nuclei) and processed for confocal imaging. Representative images from three independent experiments are shown (left and middle). Bar, 10 µm. The boxed areas were magnified and shown in red-framed images where cell edges are indicated with a white dashed line. A straight white line was used to generate the fluorescence intensity profile and for determining the percentage of colocalization, as before. Arrows point toward areas of colocalization (seen only in the EspH_1-25_-EspZ-2xHA-SBP expressing cells). Results are presented as mean ± SD. ****P < 0.0001. (**B**) Effects on lytic host cell death. LDH release measurements. HeLa cells were infected with the indicated EPEC strains and the LDH release and PI uptake assays were performed, as described in Materials and Methods section and Fig. 1C. Results are presented as mean ± SD of three or four independent measurements. ****P < 0.0001, non-significant (ns) *P* > 0.05.

To address further this, we took advantage of the loop-introduced TEV site. Initially, streptavidin beads with attached EspZ-FLAG-TEV-2xHA-SBP, or the TEV-tag lacking EspZ-2xHA-SBP, were subjected to “on-bead” digestion with recombinant TEV protease (TEVp) at 4°C, as described in the Methods. IB analysis shows complete cleavage of the EspZ-FLAG-TEV-2xHA-SBP but not the EspZ-2xHA-SBP effector (Fig. 2A, upper right). These results provide a “proof of principle” that the TEVp can cleave the loop-incorporated TEV site. “On-cell” TEVp digestion at 4°C of infected cells (see Materials and Methods section) resulted in partial cleavage of translocated EspZ-FLAG-TEV-2xHA-SBP, but not of EspZ-2xHA-SBP (Fig. 2A, lower right), suggesting that the TEV site in the EspZ loop is exposed to the TEVp in the extracellular environment. The results combined suggest that the loop of translocated EspZ is indeed exposed to the extracellular environment of the infected cells.

To analyze the possible exposure of the *C*-terminal SBP of translocated EspZ-2x-HA-SBP to the host cell cytoplasm, streptavidin (strep)-KIFC1*-mCh was ectopically expressed in HeLa cells, which were subsequently infected with EPEC-ΔespZ*/pEspZ-2xHA-SBP. If SBP contacts the host cell cytoplasm, it should interact with the cytoplasmic strep of the strep-KIFC1*-mCherry, resulting in extensive colocalization of the two proteins. Indeed, such colocalization was observed in cells infected with EPEC-ΔespZ*/pEspZ-2xHA-SBP, following EspZ labeling with anti-SBP antibodies (Fig. 2B). Lack of colocalization was observed in cells expressing strep-KIFC1*-mCherry but infected with EPEC-ΔespZ*/pEspZ-2xHA (Fig. S4). These results suggest that the *C*-terminus of translocated EspZ extends to the host cell cytoplasm.

### Unlike translocated EspZ, ectopically expressed EspZ extensively localizes to host mitochondria

Previous studies suggested that EspZ targeted to mitochondria protects against host cell death induced by EPEC-Δ*espZ* (11, 13, 14, 17, 18). In these studies, it has been shown that infection with EPEC-Δ*espZ*, but not with EPEC-Δ*espZ*/pEspZ, diminished the mitochondrial membrane potential, an activity that can be promoted by mitochondrial cell death. Additional studies have shown that ectopically expressed EspZ indeed colocalizes with mitochondrial markers (17, 20). However, the localization of translocated EspZ vis-à-vis mitochondria has not been studied. To explore this, confocal imaging was performed on HeLa cells expressing the mitochondrial marker 4xMTS-mNeonGreen and infected with EPEC-ΔespZ*/pEspZ-2xHA-SBP. Data in Fig. 3A show that a minor fraction (~2 %) of translocated EspZ colocalizes with the 4xMTS mitochondrial marker, suggesting that most of the translocated effector does not contact host mitochondria. To test the capability of ectopically expressed EspZ to target mitochondria, pcDNA expressing EspZ-2xHA-SBP was coexpressed with the mitochondrial marker in HeLa cells. Cells were then stained with anti-HA (to visualize EspZ) and DAPI (to visualize cell nuclei). Colocalization analyses of confocal images show significant (~60%) colocalization between the two markers (Fig. 3B). These data indicate that, unlike the translocated effector, ectopically expressed EspZ is indeed significantly trafficked to mitochondria.

### Ability of ectopically expressed EspZ to target mitochondria does not correlate with the capacity of the injected effector to protect against cell death

Our next aim was to examine whether a correlation exists between the ability of ectopically expressed EspZ to target mitochondria and the capacity of the translocated effector to protect against cell death. Ectopically expressed EspZ-FLAG-TEV-2xHA-SBP showed diffuse staining throughout the entire cell volume, which did not colocalize with the mitochondrial marker (Fig. 4A). These results suggest that the trafficking of EspZ-FLAG-TEV-2xHA-SBP to mitochondria is significantly impaired by the mutated extracellular loop of EspZ. Nevertheless, the LDH release and PI uptake assays showed that cell infection with the EPEC-ΔespZ*/pEspZ-FLAG-TEV-2xHA-SBP confers protection against cell death induced by EPEC-Δ*espZ* (Fig. 4B). The mutant effector was also properly translocated into the infected cells (Fig. S3B), where, as previously noted, it resides nearby the infection sites (Fig. 2A). These data suggest that while the ectopically expressed effector avoided trafficking to mitochondria, the mutant-injected effector preserved anti-cell death activities.

The *N*-terminal ~20 amino acids of EspZ are highly conserved and sufficient to direct the effector translocation (14). While the *N*-terminal cytoplasmic segment of EspZ does not contain predicted MTS, it may possess a non-canonical signal (24) involved in its trafficking to mitochondria. To investigate this, the *N*-terminal 25 amino acids of EspZ have been replaced with the *N*-terminal 25 amino acids of EspH (EspH_1-25_-EspZ-2xHA-SBP; Fig. S5A), an effector known not to be localized in host mitochondria (29, 30). Upon infection, EspH_1-25_-EspZ-2xHA-SBP is translocated into HeLa cells (Fig. S5B) and the translocated effector is localized adjacent to EPEC infection sites (Fig. S5C). Compared to EspZ-2xHA-SBP (Fig. 3B), ectopically expressed EspH_1-25_-EspZ-2xHA-SBP showed reduced colocalization levels with 4xMTS-mNeonGreen, suggesting the *N*-terminal 25 aa of the effector play a role in mitochondrial targeting. Nevertheless, cell infection with EPEC-ΔespZ*/pEspH_1-25_-EspZ-2xHA-SBP resulted in robust protection against lytic cell death induced by EPEC-ΔespZ* (Fig. 4B). These data combined suggest that the extracellular loop and the *N*-terminus of EspZ are important for localizing the ectopically expressed effector to mitochondria. Additionally, the data suggest that there is no correlation between the capacity of ectopically expressed EspZ to target mitochondria and the ability of the injected effector to protect against cell death.

### EspZ mutant consisting of the *N*-terminal cytoplasmic domain, the first predicted membrane-spanning domain and the extracellular loop fused to SBP (EspZ-74aa-SBP) fails to protect against EPEC-ΔespZ*-induced cell death

**Fig 5:**
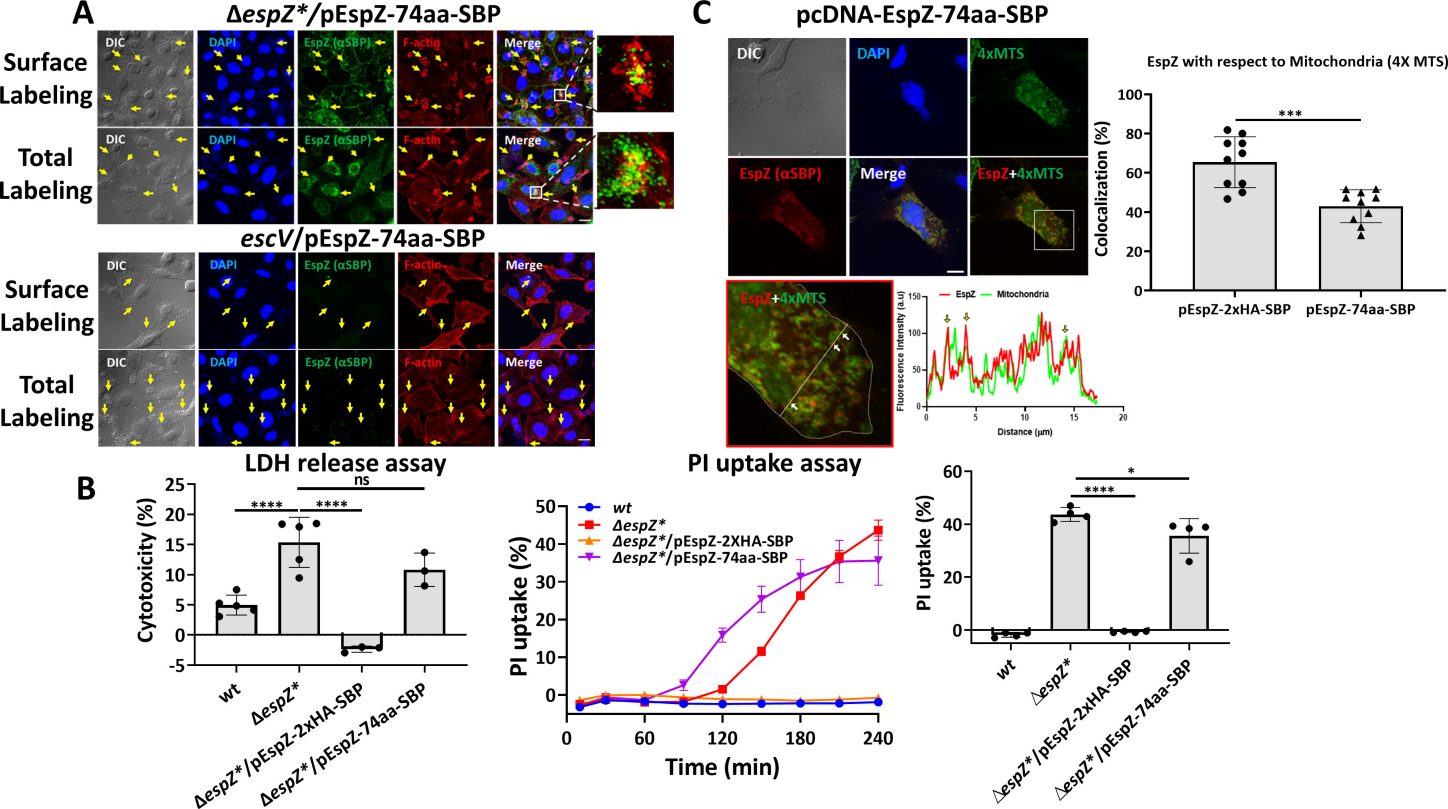
Characterization of EspZ-74aa-SBP. An EspZ mutant bearing the *N*-terminus cytoplasmic, first membrane spanning, and extracellular loop domains (illustrated in Fig. S6A) was generated and expressed in bacteria to generate EPEC-ΔespZ*/pEspZ-74aa-SBP, and which were subsequently used to infect HeLa cells, or in HeLa cells using pcDNA-EspZ-74aa-SBP encoding plasmid. (**A**) Confocal analysis of surface and total labeling. HeLa cells infected with the indicated EPEC strains were subjected to Surface and Total immunolabeling of the effector protein using anti-SBP antibodies. Images are representative of three independent experiments. Insets are used to show a small portion of the image at higher magnification. Arrows point toward cell-attached bacterial microcolonies. Bar, 10 µm. (**B**) Effects on lytic cell death. HeLa cells were infected with the indicated EPEC strains and subjected to LDH release and PI uptake measurements, as described in Materials and Methods section. Results are mean ± SD of three or four independent measurements. *P-value < 0.05, ****P-value < 0.0001, non-significant (ns) *P*-value > 0.05. (**C**) Colocalization of ectopically expressed EspZ-74aa with respect to mitochondria. HeLa cells were cotransfected with pcDNA plasmids encoding 4xMTS-NeonGreen and EspZ-74aa-SBP. The effector was immunostained with anti-SBP antibodies, and its colocalization vis-a-vis the mitochondrial marker 4xMTS was analyzed, as in Fig. 3B. Results are the mean ± SD. * *P*-value ≤ 0.5, *** *P*-value ≤ 0.001.

We next generated an EspZ mutant consisting of the *N*-terminal, the first transmembrane, and the extracellular loop fused to SBP (EspZ-74aa-SBP; illustrated in Fig. S6A). The mutant effector was translocated into HeLa cells upon infection (Fig. S6B). Surface labeling with anti-SBP antibodies of infected cells showed punctate staining delineating the cell periphery and staining that coincided with infection sites (Fig. 5A, upper; “Surface Labeling”). Immunofluorescence analysis of infected cells using the “Total Labeling” approach showed predominantly patchy perinuclear staining of the host cell cytoplasm (see Fig. 5A, upper; “Total Labeling”). The surface and total labeling of cells infected with EPEC-*escV*/pEspZ-74aa-SBP were negligible (Fig. 5A, lower), suggesting that the labeling observed in the EPEC- ΔespZ*/pEspZ-74aa-SBP-infected cells is contributed by the translocated effector. These results suggest that the translocated EspZ-74aa-SBP mutant is distributed in cellular sites that are markedly different from that of EspZ-2xHA-SBP or EspZ-FLAG-TEV-2xHA-SBP.

Interestingly, the mutant effector failed to protect against host cell death on cell infection (Fig. 5B), and the cell death effects, judged by the PI uptake assay, were not significantly affected by IPTG concentrations used to induce the effector protein expression levels (Fig. S6C). Ectopically expressed EspZ*-*74aa-SBP showed a minor effect (~40%) on colocalization levels concerning the 4xMTS mitochondrial marker (Fig. 5C). These data suggest that the *N*-terminal, first transmembrane, and the extracellular loop domains of EspZ are enough for targeting of the ectopic effector to mitochondria, yet incapable of protecting against lytic cell death on translocation. These results are also consistent with the lack of correlation between the abilities of EspZ to target mitochondria and protect against host cell death.

### Tir is important for confining EspZ localization to infection sites and for protection against host cell death

**Fig 6:**
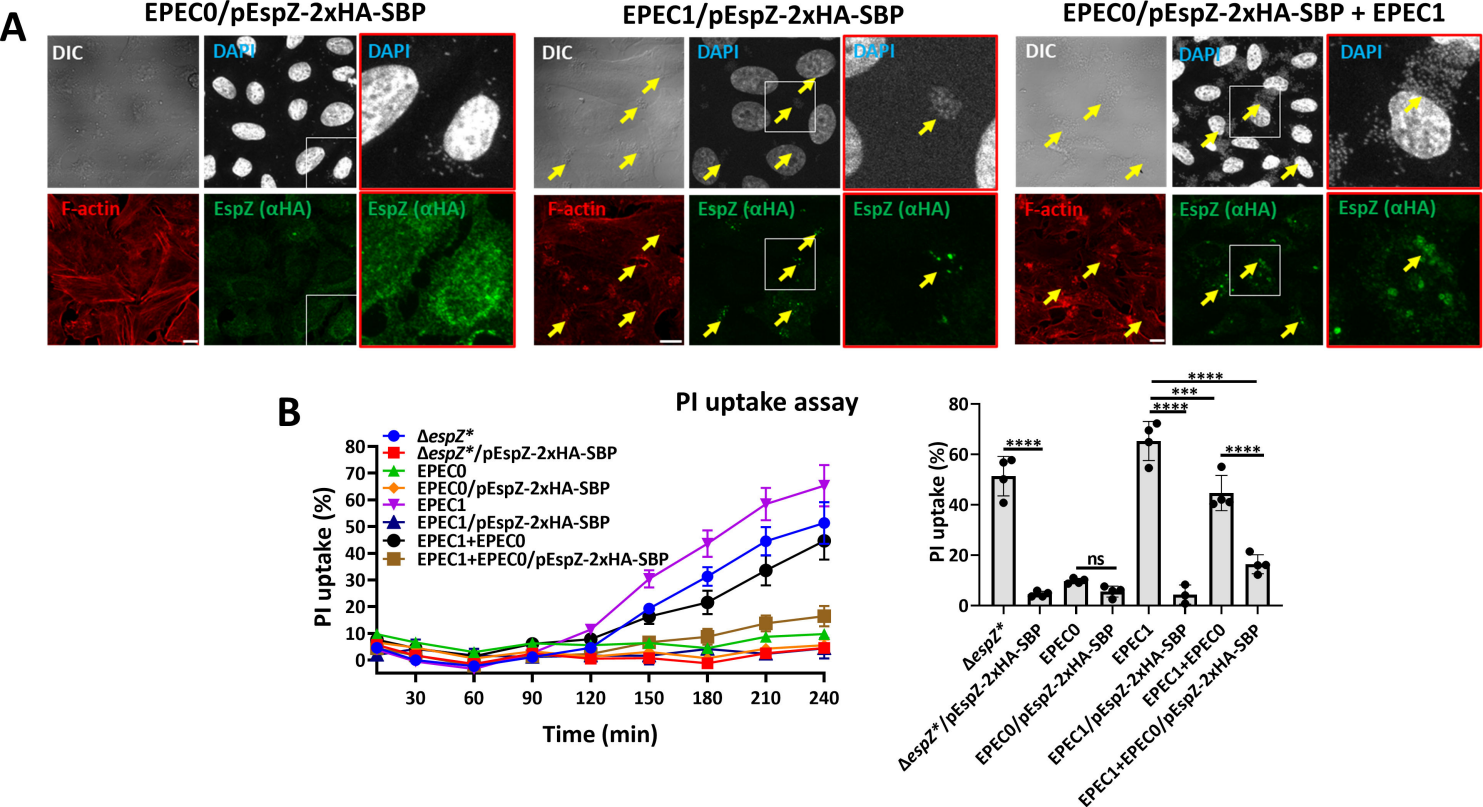
Tir confers EspZ localization at infection sites and protection against cell death. (A) Confocal analysis. HeLa cells were infected with primed EPEC0/pEspZ-2xHA-SBP (left), EPEC1/pEspZ-2xHA-SBP (middle), or EPEC0/pEspZ-2xHA-SBP + EPEC 1 (right) for 120 minutes at 37°C. Cells were fixed, permeabilized, stained with anti-HA antibodies (to visualize EspZ), DAPI (DNA), TR-phalloidin (F-actin), and processed for confocal imaging. Representative images from three experiments are shown. Red-framed images, which are enlargements of the white-boxed regions, are shown to increase visual clarity. Arrows point to infecting bacterial microcolonies. Bar, 10 µm. (B) Effects on lytic cell death. HeLa cells were infected with the indicated primed EPEC strains for 120 minutes at 37°C. The PI uptake assay was performed and analyzed, as described in Materials and Methods section. Results are the mean ± SD of four independent measurements. ***P-value < 0.001, ****P-value < 0.0001, non-significant (ns) *P*-value > 0.05.

Next, we hypothesized that EspZ-2xHA-SBP localization at infection sites is mediated by translocated Tir. If so, it would be reasonable to hypothesize that EspZ exerts its anti-cell death functions at this location. To address this, we took advantage of the following EPEC strains: EPEC0, which does not encode any type III secreted effectors, EPEC1, which expresses Tir only (21, 31), EPEC0 expressing EspZ-2xHA-SBP (EPEC0/pEspZ-2xHA-SBP), and EPEC1 expressing EspZ-2xHA-SBP (EPEC1/pEspZ-2xHA-SBP) strains. Using the effector “translocation assay,” we demonstrate that EspZ is translocated into EPEC0/pEspZ-2xHA-SBP- or EPEC1/pEspZ-2xHA-SBP-infected cells (Fig. S7). Confocal imaging of the EPEC0/pEspZ-2xHA-SBP-infected cells showed a punctate and spread over the entire cell volume localization of the translocated effector (Fig. 6A, left). Notably, adhered bacteria could not be detected in these cells, likely because of the absence of Tir, which mediates through binding intimin, intimate bacterial attachment to the host cell surface. Yet, the transient bacterial cell–host interactions were enough to enable EspZ translocation, as shown in Fig. S7. In contrast, infection with EPEC1/pEspZ-2xHA-SBP showed focal staining of EspZ nearby bacterial adherence sites (Fig. 6A, middle). Mixed infection with EPEC0/pEspZ-2xHA-SBP and EPEC1 also resulted in EspZ translocation into the host cells (Fig. S7) and localization at infection foci (Fig. 6A, right). These results suggest that Tir, the first translocated effector, is sufficient for localizing EspZ, the second translocated effector (9), at infection foci.

Next, we examined whether EspZ can protect against lytic cell death conferred by Tir. HeLa cells were infected with EPEC0, EPEC0/pEspZ-2xHA-SBP, EPEC1, EPEC1/pEspZ-2xHA-SBP, EPEC1+EPEC0, and EPEC0/pEspZ-2xHA-SBP+EPEC1. In these experiments, infections with EPEC-ΔespZ* and EPEC-ΔespZ*/pEspZ-2xHA-SBP served as negative and positive controls, respectively. Infection with EPEC0 or EPEC0/pEspZ-2xHA-SBP resulted in minimal or no effect on PI uptake over time. Conversely, cell infection with EPEC1 caused a significant time-dependent increase in PI uptake (Fig. 6B). This result agrees with previous studies suggesting that Tir induces pyroptotic cell death (21). Infection with EPEC1/pEspZ-2xHA-SBP showed minimal PI uptake levels (Fig. 6B), suggesting that the translocated effector protected against cell death caused by Tir. Mixed infection with EPEC1+EPEC0/pEspZ-2xHA-SBP resulted in low PI uptake levels compared to EPEC1+EPEC0-infected cells (Fig. 6B). The results combined suggest that EspZ translocated at infection sites protects against Tir-induced cell death.

### Translocated EspZ increases the microcolony area of host cell–adhered bacteria

**Fig 7:**
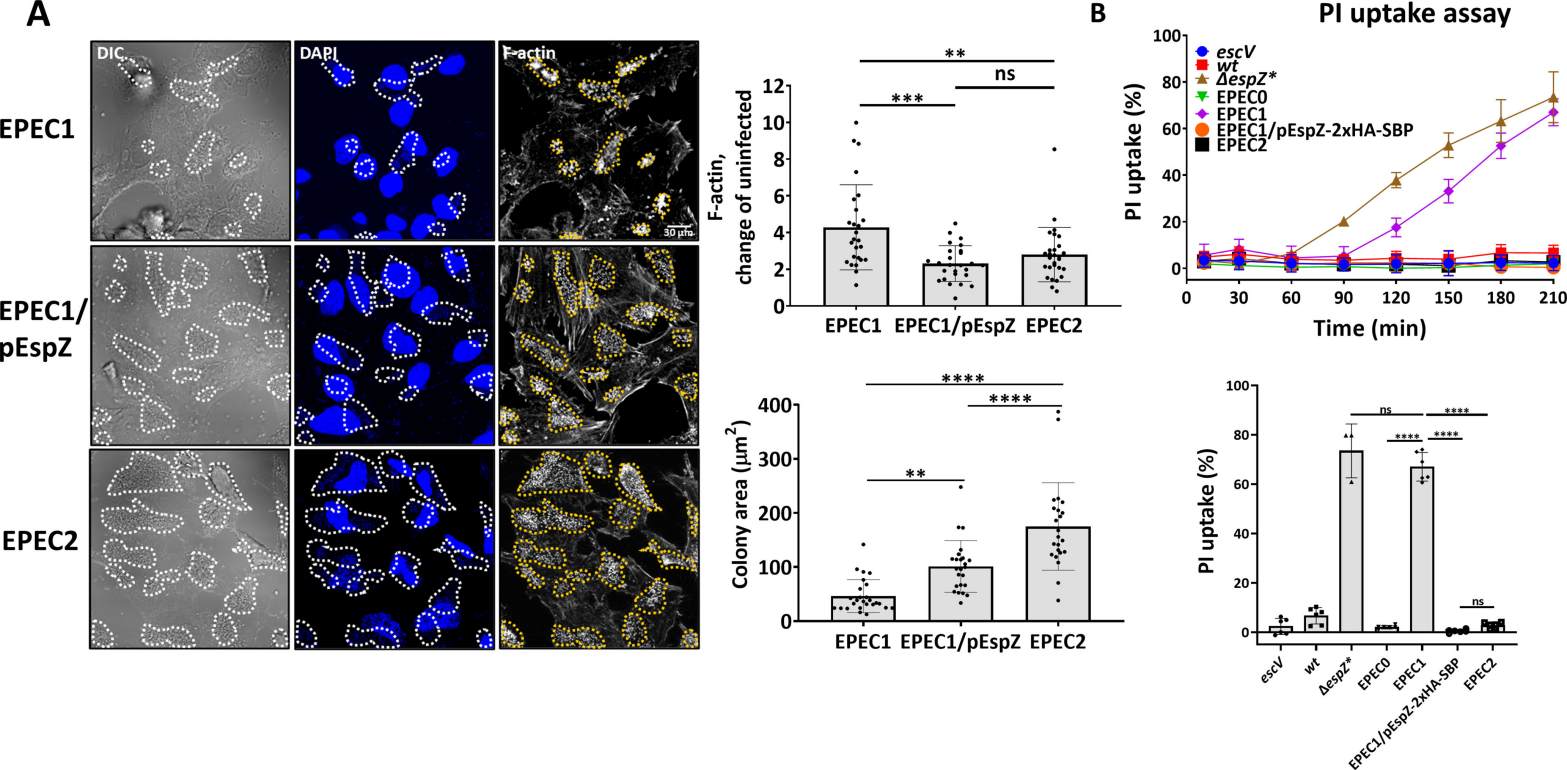
EspZ effects on Tir-induced F-actin pedestals, microcolony area, and lytic cell death of infected HeLa cells. (**A**) Effects on F-actin pedestals’ intensity and microcolony area. HeLa cells were infected with primed and indicated EPEC strains for 120 minutes at 37°C. Cells were then fixed, permeabilized, and stained with DAPI (DNA), and TR-phalloidin (F-actin), and imaged by confocal microscopy. Representative images (out of three experiments) are shown (left). Bar, 30 µm. Cell-attached bacterial microcolonies are marked with a dotted line. The TR-phalloidin fluorescence intensity levels at infection sites, which represent F-actin-rich pedestals, were measured by Fiji (NIH) and normalized to fluorescence background levels of uninfected cell regions, as described (32). Results are presented as mean ± SD for measurements performed on ~20 microcolonies (upper right) also determined the area of each marked microcolony (bottom right). *P-value ≤ 0.05, **P-value ≤ 0.01, ***P-value ≤ 0.001, ****P-value ≤ 0.0001, non-significant (ns) *P*-value > 0.05. (**B**) Effects on lytic cell death. Cells were infected with the indicated EPEC strains, and the PI uptake assay was performed, as described in Materials and Methods. ****P-value < 0.0001, non-significant (ns) *P*-value > 0.05.

Previous studies suggested that EspZ, either ectopically expressed or translocated, blocks EPEC-induced host cell death by delaying the translocation of type III secreted effectors that promote cell death, for example, Tir, EspF, and Map (11). Tir–intimin interactions are essential for F-actin-rich pedestal generation (7, 8). Hence, EspZ-mediated inhibition of Tir translocation should diminish pedestal formation. As expected, cells infected with EPEC1 (expressing Tir only) led to the appearance of F-actin-rich pedestals in nearly all infection sites (Fig. 7A). Cell infection with EPEC2 (expressing Tir and EspZ) also generated pedestals, but with somewhat lower F-actin fluorescence intensity (Fig. 7A). Cell infection with EPEC1/pEspZ yielded a similar result (Fig. 7A). Despite the minor effects, these results may agree with the hypothesis that translocated EspZ inhibits the translocation of Tir through inhibiting the T3SS (11). Alternatively, EspZ may attenuate the capacity of translocated Tir to mediate actin polymerization. Both scenarios will result in reduced pedestal generation.

Studies have shown that F-actin-rich pedestal formation is essential for bacterial attachment, growth, and colonization of the host (3, 33 - 36). If indeed so, one would expect that translocated EspZ would inhibit not only pedestal formation but also the microcolony size of cell-adhered bacteria. Measurements of the cell-associated microcolony area showed that this may not be the case, as that area was significantly increased in EPEC1/pEspZ-2xHA-SBP- or EPEC2-, compared to EPEC1-infected cells (Fig. 7A). Notably, EPEC2 microcolony area was greater than EPEC1/pEspZ-2xHA-SBP. This could be attributed to the effects of protein expression levels or differences in the interplay between the endogenous or exogenously expressed EspZ with translocated Tir. Nevertheless, these results agree with the hypothesis that translocated EspZ enhances host colonization by the microbe. Similar results were obtained in Caco2BBe (intestinal epithelial)-infected cells (Fig. S8), suggesting that the effect is not cell-type specific. Importantly, EPEC2 and EPEC1/pEspZ-2xHA-SBP had a profound effect in protecting the cells against host cell death promoted by EPEC1 (Fig. 7B; Fig. S8B), suggesting that EspZ boosts host cell viability and colonization.

## DISCUSSION

EspZ is one of the four type III secreted effectors (EspZ, Tir, EspF, and NleA) suggested to be essential for eliciting a disease by A/E pathogens in mice and rabbit models (12, 13, 37). It is the first translocated effector after Tir (10), and as Tir has been implicated in inducing pyroptotic cell death (21, 22), the anti-death activity of EspZ could be crucial for establishing bacterial colonization of the host at an early infection time. Therefore, a better understanding of the molecular mechanisms underlying the EspZ capacity to inhibit host cell death is critical for better understanding the mechanisms by which A/E pathogens induce a disease.

Several studies have related the capability of EspZ to protect against host cell death to its ability to target host mitochondria. Those include (i) interactions of EspZ with the mitochondrial translocator (e.g., TIM17b) (17), (ii) targeting mitochondrial proteins which control mitochondrial integrity (e.g., FIS1) (20), and (iii) blocking the translocation of pro-death effectors, such as EspF and Map (11, 24, 29). However, EspZ has been localized to mitochondria only when the effector was ectopically expressed in mammalian cells, leaving the mitochondrial localization of the translocated effector, which is the more physiologically relevant form of the effector, unexplored.

Here we show that translocated EspZ is confined to plasma membrane infection sites (Fig. 1A and Fig. 2A), with its predicted extracellular loop facing the extracellular environment (Fig. 2A), and the *C*- (and most likely the *N-*) termini facing the host cell cytoplasm (Fig. 2B). Translocated EspZ displayed minimal, or no, colocalization with host mitochondria (Fig. 3), while consistent with previous studies, the ectopically expressed effector localized effectively with mitochondria (Fig. 3B). These data hence argue that mitochondria are not major host sites targeted by translocated EspZ. EspZ localization at infection sites is dictated by translocated Tir (Fig. 6A), and this correlates with the effector’s ability to protect against Tir-mediated cell death (Fig. 6B). Since Tir is thought to be translocated into the host cell plasma membrane, it would be conceivable to hypothesize that the plasma membrane and communicating endomembranes are the sites for EspZ action.

Moreover, we show that there is no correlation between the ability of ectopically expressed EspZ to reach mitochondria and the capacity of the translocated effector to protect against cell death (Fig. 3, 4, 5). Of course, these observations do not exclude the possibility that the translocated effector performs its anti-death function by targeting mitochondrial proteins located outside mitochondria. Mitochondria as well as other organelles, for example, the endoplasmic reticulum, have been reported to contact the plasma membrane, and some of these interactions have been suggested to modulate cell death (38 - 40). Hence, EspZ may target these mitochondrial interfaces to protect against cell death, while not showing clear colocalization with the organelles. Studies have also suggested that EspZ targets proteins other than mitochondrial proteins, for example, the host CD98 protein that contributes to protection against EPEC-mediated cell death (19), as well as components of the bacterial T3SS (11). However, a comprehensive mechanistic view of how EspZ controls host cell death via these or other interactions is still lacking and awaits further investigation at the subcellular and molecular levels.

## MATERIALS AND METHODS

### EPEC strains, antibodies, plasmids, primers, and reagents

The EPEC strains, primary and secondary antibodies, plasmids, primers, and gBlocks are listed in Tables S1 to S4, respectively. Notably, the EPEC-ΔespZ* mutant strain was constructed in the background of the IE6 and PP4 deleted islands. These two genomic islands encode effectors counteracting host cell death (e.g., *nleB*, *nled*, and *nleH*; reviewed in reference [23]). Thus, deleting these genes is expected to minimize the potential anti-host cell death effects elicited by these effectors. The recombinant plasmids were constructed by amplifying the insert and vector using respective primers and the Platinum-SuperFi Master Mix protocol (Thermo Scientific, no. 12358-010). For ligating the insert with their respective vectors, the standard Gibson assembly method (41) and the Gibson assembly master mix (NEB, no. E2611) were used according to the manufacturer’s instructions. Bacterial colonies containing the recombinant plasmids were selected on a Luria broth (LB) agar plate with relevant antibiotics (ampicillin [100 µg/mL], kanamycin [50 µg/mL], streptomycin [50 µg/mL], and nalidixic acid [30 µg/mL]). Nucleotide sequences of all constructs were confirmed by the Genomic Technologies Facility (https://www.bio.huji.ac.il/en/units_the_national_center_for_genomic_technologies), using SANGER sequencing.

### Cloning of EspZ constructs into a bacterial vector

#### Construction of pSA10-EspZ-2xHA-SBP

The pSA10-EspZ-2xHA-SBP plasmid (the pSA10 plasmid has been described in Table S3 and reference [42]) was constructed by replacing the EspH-6xHis of pSA10-EspH-6xHIS-SBP with EspZ-2xHA. Oligonucleotides 1F' and 2R' were used to obtain the PCR-linearized pAA6284 vector; oligonucleotides 3F' and 4R' were used to PCR amplify EspZ from the pEspZ-mCherry template; and oligonucleotides 5F' and 6R' were used to PCR amplify 2xHA from gBlock 7 template. A schematic illustration of the protein is presented in Fig. S1.

#### Construction of pSA10-EspZ-2xHA

The pSA10-EspZ-2xHA plasmid was constructed by replacing EspH-6xHis-SBP of pAA6284 with EspZ-2xHA. Oligonucleotides 8F' and 2R' were used to produce the PCR-linearized pAA6284 vector. Oligonucleotides 3F' and 4R' were used to PCR amplify EspZ from the mCherry-EspZ template, and oligonucleotides 5F' and 9R' were used to PCR amplify 2xHA from gBlock 7 template.

#### Construction of pSA10-EspZ-FLAG-TEV-2xHA-SBP

The pSA10-EspZ-FLAG-TEV-2xHA-SBP plasmid was constructed by adding the sequence encoding 1xFLAG-1xTEV (with glycine-glycine-serine (GGS) linker upstream and downstream to it) to the EspZ loop (after Asp69) of pSA10-EspZ-2xHA-SBP. Oligonucleotides 10F' and 11R' were used to insert this fragment into the pSA10-EspZ-2xHA-SBP vector.

#### Construction of pSA10-EspH_1-25_-EspZ-2xHA-SBP

The pSA10-EspH_1-25_-EspZ-2xHA-SBP plasmid was constructed by replacing EspZ_1-25_ of pSA10-EspZ-2xHA-SBP with EspH_1-25_. Oligonucleotides 12F' and 13R' were used to obtain the PCR-linearized pSA10-EspZ-2xHA-SBP vector, and oligonucleotides 14F' and 15R' were used to PCR amplify EspH_1-25_ from gBlock 16 template.

#### Construction of pSA10-EspZ-74aa-SBP

The pSA10-EspZ-74aa-SBP plasmid was constructed by deleting 45 aa (75–119), namely, removing 24 aa of EspZ, the subsequent 2xHA tags, and 3 aa of the linkers from the original pEspZ-2xHA-SBP encoding plasmid (Fig. S1). This has resulted in tagging of EspZ at aa 74 with a GGS linker and subsequent SBP, as illustrated in Fig. S6A. Oligonucleotides 1F' and 22R' were used to obtain the new PCR-linearized vector based on the pSA10-EspZ-2xHA-SBP template, and ligation was done by Gibson assembly.

### Cloning of EspZ constructs into a mammalian vector

The pcDNA(3.1)-EspZ-2xHA-SBP, pcDNA(3.1)-EspZ-FLAG-TEV-2xHA-SBP, pcDNA(3.1)-EspH_1-25_-EspZ-2xHA-SBP, and pcDNA(3.1)-EspZ-74aa-2xHA-SBP plasmids were constructed by replacing the GFP (1-10) of pcDNA3.1-GFP (1-10) with the relevant fragment from pSA10-EspZ-2xHA-SBP, pSA10-EspZ-FLAG-TEV-2xHA-SBP, pSA10-EspH_1-25_-EspZ-2xHA-SBP, and pSA10-EspZ-74aa-2xHA-SBP, respectively. Oligonucleotides 17F' and 18R' were used to obtain the PCR-linearized pcDNA3.1-GFP (1-10) vector, and oligonucleotides 19F' and 20R' were used to PCR amplify EspZ-2xHA-SBP and EspZ-FLAG-TEV-2xHA-SBP from their templates. For pcDNA3.1-EspH_1-25_-EspZ-2xHA-SBP, oligonucleotides 19F' and 21R' were used to obtain the PCR-linearized pcDNA3.1-GFP (1-10) vector, and oligonucleotides 14F' and 20R' were used to PCR amplify EspH_1-25_-EspZ-2xHA-SBP from its template. For pcDNA3.1-EspZ-74aa-2xHA-SBP, oligonucleotides 23F' and 24R' were used to PCR amplify EspZ-74aa-2xHA-SBP from its template.

### Cell culture

HeLa and Caco-2_BBe_ cells were cultured, as before (32, 43 - 45).

### Bacterial pre-activation (priming) and cell infection

An overnight LB EPEC culture was diluted 1:50 (vol/vol) with pre-equilibrated (37°C, 5% CO_2_, 95% humidity) Dulbecco's modified eagle medium (DMEM)-high glucose for 3 hours in a CO_2_ incubator. When effector protein expression had to be induced, isopropyl β-D-1 thiogalactopyranoside (IPTG; Promega no. V395D; 0.2 mM, unless otherwise indicated) was added to the activation medium after 2.5 hours of incubation. HeLa cells were cultured to 70%–80% confluency on a 24-well plate (NUNC-Cell-Culture Treated Multidishes; no. 142475; Thermo Scientific) or 6-well plate (NUNC-Cell-Culture Treated Multidishes), placed on a heating block (37°C), and washed three times with pre-warmed (37°C) plain DMEM. After the last wash, the medium was replaced with the pre-activated EPEC-containing medium and incubated in the CO_2_ incubator for the indicated infection times.

### Lactate dehydrogenase (LDH) release assay

HeLa cells were seeded in a 24-well plate (0.8 × 10^5^ cells/well) and incubated for 48 hours in a CO_2_ incubator (37°C, 5% CO_2_, 90% humidity) until reaching 70%–90% confluency. Cells were then infected with pre-activated EPEC strains for 60 minutes at 37°C. Media bathing the cells were collected and centrifuged (600× *g*, 10 minutes), and the LDH released into them was measured by the LDH-Cytotoxicity Assay (Abcam no. ab65393), as described in the manufacturer’s protocol. The % LDH release was calculated as follows,


$$Cytotoxicity\;(\%)=\frac{Test\;Sample-Low\;control}{High\;control-Low\;control}\times 100,$$

where the “Test Sample” is the infected cells, “Low control” is the uninfected cells, and “High control” is the uninfected cells lysed in the lysis solution.

### Propidium iodide (PI) uptake assay

The PI uptake assay was applied as described (21), with some modifications. HeLa cells (0.5 × 10^4^ cells/well) were seeded in a black clear-bottom 96-well plate (Greiner Bio-One, no. 655090) 1 day before infection. Cells were then washed and incubated with phenol-red-free DMEM (high glucose; Biological Industries, no. 01-053-1A) for 15 minutes before infection. Bacteria were primed in phenol-red-free DMEM for 3 hours. IPTG was added after 2.5 hours at the indicated concentrations. PI (5 µg/mL; Sigma, no. P4170) was added to the activated bacteria. Cell medium was replaced with the PI-containing activated bacteria and incubated at 37°C; 5% CO_2_. The PI fluorescence was measured (BioTek Synergy H1 plate reader; 520-nm excitation and 620-nm emission wavelengths) every 30 minutes. PI-containing plain media served as blanks. Cells that were not exposed to the bacteria, but otherwise treated equally were designated as “uninfected.” Positive controls were determined by solubilizing cells in 0.1% Triton X-100 (J.T Baker, X198-07) containing PI (5 µg/mL). The cells were immediately subjected to fluorescence measurements taken in 30-minute intervals. Peak levels were averaged and used as the “positive control” values. Following subtracting the blank from each reading, PI uptake (%) was calculated as:


$$\frac{Infected-Uninfected}{Positive\;control}\times 100$$

### Effector translocation assay

Approximately 2 × 10^5^ cells/well were seeded on three wells of a six-well plate for 48 hours until reaching 80%–90% confluency. The cells were then infected with EPEC-ΔespZ* expressing EspZ strains for 60 minutes at 37°C. Infection with EPEC-*escV* expressing EspZ was used for evaluating the T3SS dependence of effector translocation. Following infection, cells were washed three times with ice-cold phopshate-buffered saline (PBS) and lysed in 60 µL of ice-cold solubilization buffer (PBS supplemented with 1% of Triton X-100 and 0.5% of protease and phosphatase inhibitor). Following 3-minute incubation on ice, the lysate was pipetted up and down and then centrifuged (10,000× *g*, 4°C, 10 minutes). The supernatant (detergent soluble, S) and pellet (detergent-insoluble, IS) were isolated. The pellet was resuspended in 60 µL of the lysis buffer. Twenty microliters of 4× SDS-PAGE sample buffer were added to the S and IS fractions. The samples were then heated (95°C, 10 minutes) and analyzed by SDS-PAGE, followed by western blotting. The presence of EspZ in the fractions was detected with anti-HA antibodies or anti-SBP antibodies. The lysate protein load was assessed using anti-α-Tubulin (αα-Tubulin) or anti-GAPDH antibodies. The assumption is that the host cells are susceptible to solubilization by the detergent, whereas the cell-associated bacteria remain largely insoluble. As effector translocation is expected to be T3SS dependent, we expect that the effector in cells infected with EspZ expressed in EPEC-*escV* (e.g., EPEC-*escV*/pEspZ-2xHA-SBP) will be detected mainly in the detergent IS fraction, while in cells infected with EPEC-ΔespZ*/pEspZ-2xHA-SBP, a significant fraction of the expressed effector will also be detected in the detergent S fraction as a result of effector translocation into the host cells. Effector translocation (%) was calculated as:


$$\frac{Effector\;\left(S\right)}{Effector\;\left(S\right)+Effector\;\left(IS\right)}\times 100$$

### SDS-PAGE and immunoblotting (IB)

SDS-PAGE and western blotting were performed, as described (29). Briefly, samples were mixed with sample buffer (40% glycerol; 12% SDS; 0.2M Tris-HCl, pH 6.8; and 100 mM dithiothreitol) supplemented with bromophenol blue. The lysate was then heated at 95°C for 10 minutes, and the proteins were analyzed by SDS-PAGE apparatus (Bio-Rad Mini-PROTEAN Tetra system; 40 mA, 30 minutes), transferred to nitrocellulose membrane (Bio-Rad Trans-Blot Turbo; 2.5 A, 10 minutes), and blocked with TBST (10 mM Tris, pH 8.0; 150 mM NaCl; and 0.1% Tween-20) + 2.5% bovine serum albumin (BSA) + 1% milk. Membranes were probed with respective antibodies diluted in TBST + 5% BSA either for 1 hour at 22°C or overnight at 4°C with gentle rocking. Membranes were imaged using a Fusion FX spectra imager (Vilber Smart Imaging, Collègien, France), and the protein band intensity was measured by Fiji (National Institutes of Health [NIH]).

### Ectopic expression by transient transfection

HeLa cells (0.8 × 10^5^ cells/well) were seeded on coverslips (12 mm Φ; Microscope Cover Glasses; no. 0111520) placed in a 24-well plate. Following 12 hours, cells were washed two times with pre-warmed (37°C) Dulbecco’s PBS (Biological Industries). The buffer was replaced with 450 µL of DMEM without antibiotics ([DMEM-High-Glucose, SARTORIUS] supplemented with 10% fetal bovine serum [FBS] [Qualified FBS, Gibco] and 1% GLN [L-glutamine, SARTORIUS]). A “transfection mixture” was prepared by adding 500 ng of the relevant plasmid to 50 µL of Opti-MEM I (1×) (Gibco, no. 31985-047), pipetting up and down 10 times followed by the addition of 1.5 µL of TransIt-X2 solution (MIRUS, no. MIR 6004). The “transfection mixture” was then mixed again, incubated at 22°C for 20 minutes, and then added to the cells in 450 µL DMEM complete media without antibiotics. Cells were then incubated for 4–5 hours in the CO_2_ incubator (37°C, 5% CO_2_, 95% humidity). Thereafter, the medium was aspirated and replaced with a fresh growth medium. Cells were analyzed ~24 hours after transfection.

### Surface and total immunolabeling of EspZ

#### Surface labeling

HeLa cells (0.8–1.2 × 10^5^ per well) were seeded in a glass coverslip placed in a 24-well plate and cultured for 48 hours (37°C, 5% CO_2_, 95% humidity). Cells were infected with EPEC, washed three times with ice-cold (4°C) PBS (137 mM NaCl, 2.7 mM KCl, 10 mM disodium phosphate [Na_2_HPO_4_], 1.7 mM potassium dihydrogen phosphate [KH_2_PO_4_], pH = 7.4) supplemented with 10% BSA and exposed simultaneously to mouse anti-SBP (0.5 µg/mL) and rabbit anti-FLAG antibodies (2.5 µg/mL) for 60 minutes at 4°C. Cells were then washed three times with ice-cold PBS and fixed with 4% paraformaldehyde (PFA 16% solution; EMS, no. 15710) for 1 hour at 4°C, washed again three times with PBS, and labeled with Alexa Fluor488 anti-mouse and Cy5 anti-rabbit secondary antibodies in PBS supplemented with 10% BSA for 40 minutes at 22°C. Cells were permeabilized with a blocking solution (PBS containing 3% vol/vol FCS, 0.1% wt/vol saponin [quillaja bark; Sigma Lifesciences, no. S7900]) for 30 minutes at 37°C and stained with Texas-Red-phalloidin (TR-phalloidin; 1:400; Life Technologies, no. T7471) in blocking solution for 40 minutes at 37°C for labeling the host cell F-actin. Cells were then washed three times with PBS, fixed with 4% PFA for 25 minutes at 22°C, washed two times with PBS, and then incubated in a DAPI-containing solution (20 µg/mL in PBS; Sigma, no. D9542) for 5 minutes at 22°C for staining DNA in host cell nuclei and bacterial microcolonies. Cells were then washed once with PBS, two times with water, and mounted on slides (76 × 26 × 1 mm; MARIENFELD company, no. 1000200), using Immu-mount (SHANDON; Thermo Scientific, no. FIS9990402).

#### Total labeling

“Total labeling” refers to the conventional immunofluorescence labeling of permeabilized cells procedure, performed as previously described (32). HeLa cells (0.8–1.2 × 10^5^) were seeded on a glass coverslip placed in a 24-well plate and cultured for 48 hours in a CO_2_ incubator. Then, cells were washed three times with pre-warmed (37°C) PBS and fixed with 4% PFA in PBS for 25 minutes at 22°C. Cells were then blocked with blocking solution for 30 minutes at 37°C. The blocking solution was replaced with a pre-heated blocking solution supplemented with anti-SBP or anti-FLAG primary antibodies. Cells were then incubated in a moisturized chamber for 60 minutes at 37°C, washed three times with the blocking solution, and incubated with an appropriate fluorescently labeled secondary antibody. TR-phalloidin was included with the secondary antibody solution to label F-actin. Cells were washed two times with the blocking solution and once with PBS. The PBS solution was replaced with a DAPI containing PBS for 5 minutes at 22°C to stain host cell nuclei and bacterial microcolonies. Cells were washed three times with PBS and fixed again with 4% PFA for 25 minutes at 22°C. Finally, cells were washed once with PBS, two times with water, and mounted on slides using Immu-Mount.

### Fluorescence microscopy and colocalization analyses

Cells were mounted and imaged using an Olympus FV-1200 laser scanning confocal microscope equipped with a 60× oil immersion objective (numerical aperture, 1.42) in a sequential mode to avoid bleed-through of fluorophore emissions. The excitation wavelengths were 405 nm, 488 nm, 561 nm, and 635 nm, and the emission filter passbands were 430–470 nm, 500–540 nm, 570–620 nm, and 640–700 nm for DAPI, green, red, and far-red fluorescence, respectively. Confocal sections were acquired at *z*-axis intervals of 0.5 µm. The images were analyzed in Fiji (NIH) (32). A maximal-intensity projection was generated for each stack. For colocalization analyses, 10–20 line intensity profiles were generated, and colocalization analysis was performed, as described (29, 44). Briefly, a line was drawn across a randomly chosen confocal section showing costaining, and the fluorescence intensity along the line of the two different channels was generated using the intensity profile tool (plot-profile plug-in) of Fiji (NIH). Colocalized labeling was scored when the fluorescence intensity copeaked at a given place along the line. Notably, images taken for colocalization analysis were acquired under identical conditions. Data are presented as percentages of colocalizing fluorescence intensity 100–150 peaks derived from the 10–20-line intensity profiles. The intensity of F-actin-rich pedestals was determined by Fiji (NIH), as described (32).

### EspZ-FLAG-TEV-2xHA-SBP cleavage with TEV protease (TEVp)

#### On-bead digestion

HeLa cells cultured on a 10-cm dish to ~80% confluency was infected with EPEC-ΔespZ*/pEspZ-2xHA-SBP or with EPEC-ΔespZ*/pEspZ-FLAG-TEV-2xHA-SBP for 60 minutes at 37°C. Cells were then washed three times with ice-cold PBS. Cells were lysed with 120-µL lysis buffer (50 mM Tris [pH 7.4], 150 mM NaCl, 0.5% NP-40) supplemented with protease and phosphatase inhibitors (Sigma, no. PPC1010-1 ML), spun (10,000× *g*, 10 minutes, 4°C), and the supernatant was exposed to 40 µL of pre-washed streptavidin agarose beads (Sigma-Aldrich, cat no. 1003487351) for 3 hours at 4°C with end-to-end rotation. Beads were washed five times, with ice-cold lysis buffer, and resuspended in 60 µL of lysis buffer. The resuspended beads were then divided into fractions (30 µL each). One fraction was treated (+) with 10 µg/mL of the recombinant purified TEVp (expressed using pRK793 plasmid kindly provided by Dr. David Waugh and purified as previously described [46]) for 20 hours at 4°C, and the second fraction was left untreated (−). Beads were spun (3,000× *g*, 1 minute, 4°C), washed three times with lysis buffer, and dried beads were incubated with SDS-PAGE sample buffer for 5 minutes at 95°C. TEV protease is expected to cleave at the ENLYFQ↓G sequence (see Fig. S3A), splitting the effector protein into two polypeptides (aa 1–89 and aa 90–183), having approximately the same molecular mass (~11 kDa). EspZ was identified following SDS-PAGE (4%–20% Bio-Rad stain-free gradient gels [cat no. 4568096]) and IB probed with anti-HA or anti-SBP antibodies.

#### On-cell digestion

Approximately 2 × 10^5^ HeLa cells/well cultured on three wells of a six-well plate for 48 hours until reaching 80% confluency. Cells were then infected with EPEC-ΔespZ*/pEspZ-2xHA-SBP or EPEC-ΔespZ*/pEspZ-FLAG-TEV-2xHA-SBP for 60 minutes at 37°C. Following infection, cells were treated with 10 µg/mL of tetracycline and 100 µg/mL of gentamycin for 1 hour at 37°C. Thereafter, the cells were washed three times with ice-cold PBS and incubated overnight at 4°C with PBS supplemented with 10 µg/mL of the recombinant purified TEVp (+) or left untreated (−). Cells were then washed three times with ice-cold PBS and lysed in 120 µL of lysis buffer supplemented with protease and phosphatase inhibitors, as above. Lysates were centrifuged (10,000× *g*, 10 minutes, 4°C), and EspZ was precipitated from the supernatant using 30-L streptavidin agarose beads, washed with lysis buffer, and attached proteins were eluted by incubating the beads in 40 µL 1× Laemmli sample buffer (0.025 M Tris-PO4 [pH 6.8], 1% β-mercaptoethanol, 0.001 M EDTA [pH 6.8], 10% glycerin, and 0.01% bromophenol blue) for 2.5 hours at 22°C with constant shaking. EspZ was identified by SDS-PAGE followed by IB.

### Statistical calculations

All statistical tests were performed on at least three independent experiments, except for data presented in Fig. 7 and Fig. S8, where the number of cells was used for statistics. We used one-way ANOVA followed by Tukey’s or Bonferroni’s multiple comparison test to determine the statistical significance when multiple groups were compared, and two-tailed unpaired Student’s *t*-test when two groups were compared. Statistical tests and graphs were made with the GraphPad Prism v. 8.4.3 software (GraphPad, San Diego, CA, USA).

## AFTER EPUB

[This article was published on 21 June 2023 with a missing word in the abstract. The abstract was corrected in the current version, posted on 30 June 2023.]

## SUPPLEMENTAL MATERIAL

Supplemental Figures mbio.00752-23-s0001.pdf Fig. S1 to S8.

Supplemental Tables mbio.00752-23-s0002.pdf Tables S1 to S4.

Supplemental Legends mbio.00752-23-s0003.docx Legends to Fig. S1 to S8.

ASM does not own the copyrights to Supplemental Material that may be linked to, or accessed through, an article. The authors have granted ASM a non-exclusive, world-wide license to publish the Supplemental Material files. Please contact the corresponding author directly for reuse.
