# Supplementary material for: Topology and function of translocated EspZ
Source: mBio. 2023 Jun 21;14(4):e00752-23. doi: 10.1128/mbio.00752-23 (PMC10470495; doi:10.1128/mbio.00752-23)
Supplement: Supplemental Figures — Fig. S1 to S8. [file mbio.00752-23-s0001.pdf]

Fig. S1

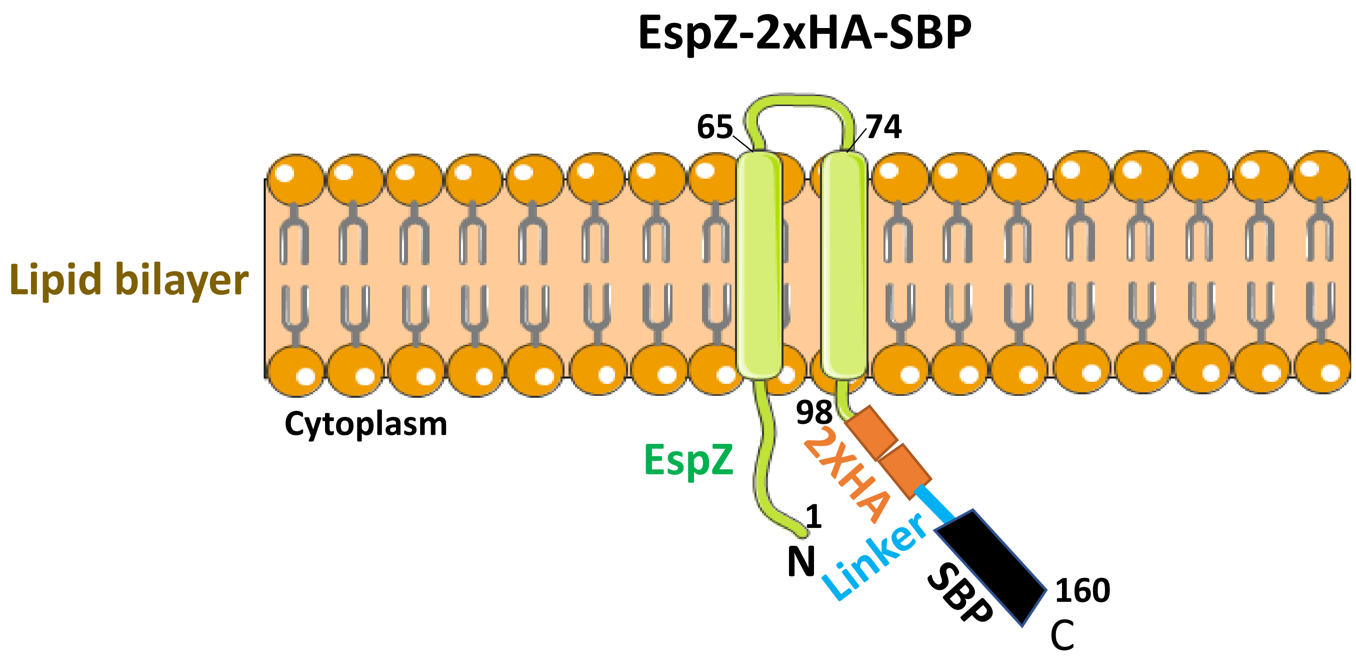

98
LAMKYAYPYDVPDYAYPYDVPDYAGGSGGSMDEKTTGWRGGHVVEGLAGELEQLRARLEHHPQGQREP 160

EspZ 2xHA Linker SBP

Fig. S2

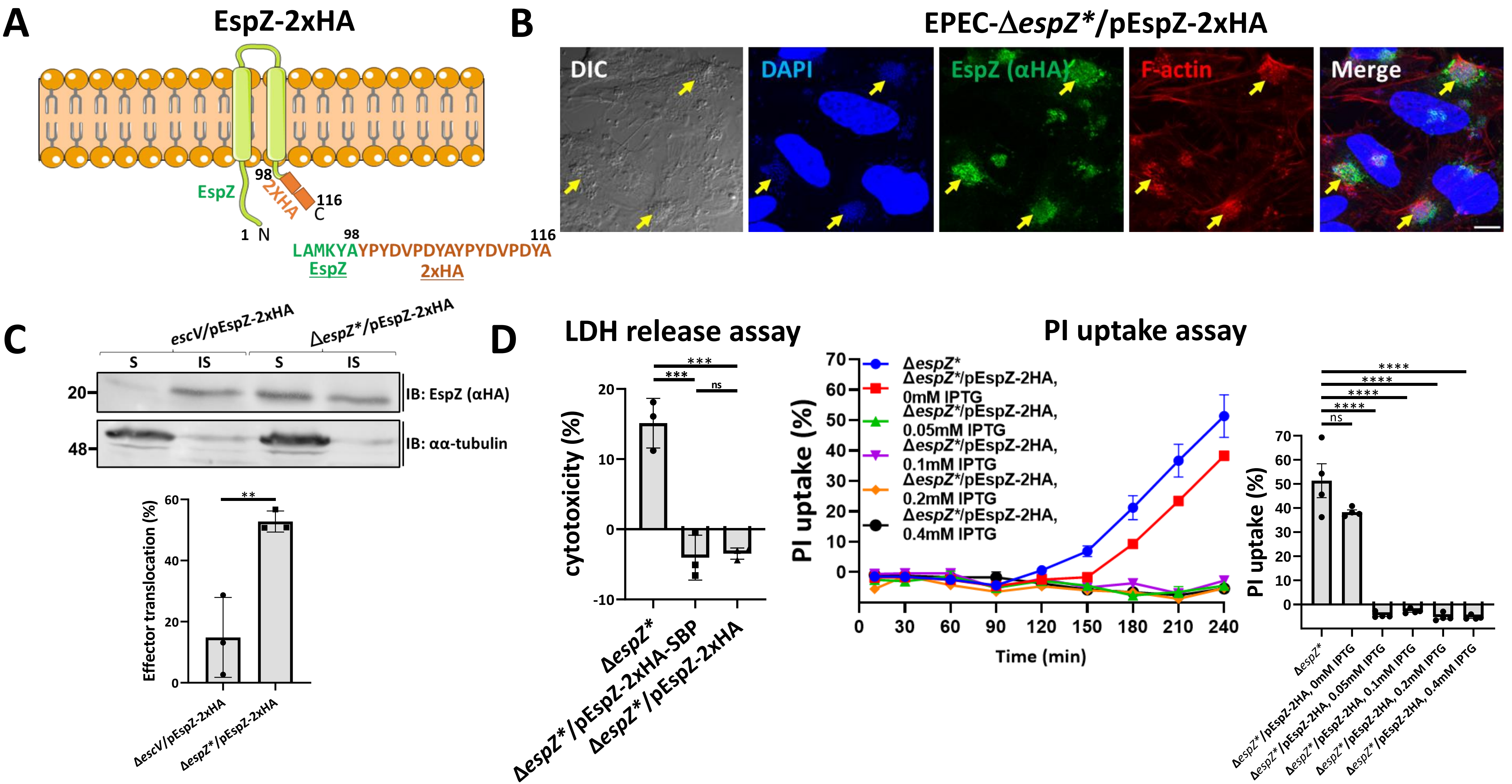

A
EspZ-2xHA
98
EspZ
2XHA
116
C
1 N
98
116
LAMKYAYPYDVPDYAYPYDVPDYA
EspZ
2xHA
B
EPEC-ΔespZ*/pEspZ-2xHA
DIC
DAPI
EspZ (αHA)
F-actin
Merge
C
escV/pEspZ-2xHA
ΔespZ*/pEspZ-2xHA
S
IS
S
IS
20
48
IB: EspZ (αHA)
IB: αα-tubulin
Effector translocation (%)
**
ΔescV/pEspZ-2xHA
ΔespZ*/pEspZ-2xHA
D
LDH release assay
cytotoxicity (%)
***
***
ns
ΔespZ*
ΔespZ*/pEspZ-2xHA-SBP
ΔespZ*/pEspZ-2xHA
PI uptake assay
PI uptake (%)
ΔespZ*
ΔespZ*/pEspZ-2HA, 0mM IPTG
ΔespZ*/pEspZ-2HA, 0.05mM IPTG
ΔespZ*/pEspZ-2HA, 0.1mM IPTG
ΔespZ*/pEspZ-2HA, 0.2mM IPTG
ΔespZ*/pEspZ-2HA, 0.4mM IPTG
Time (min)
****
ns

Fig. S3

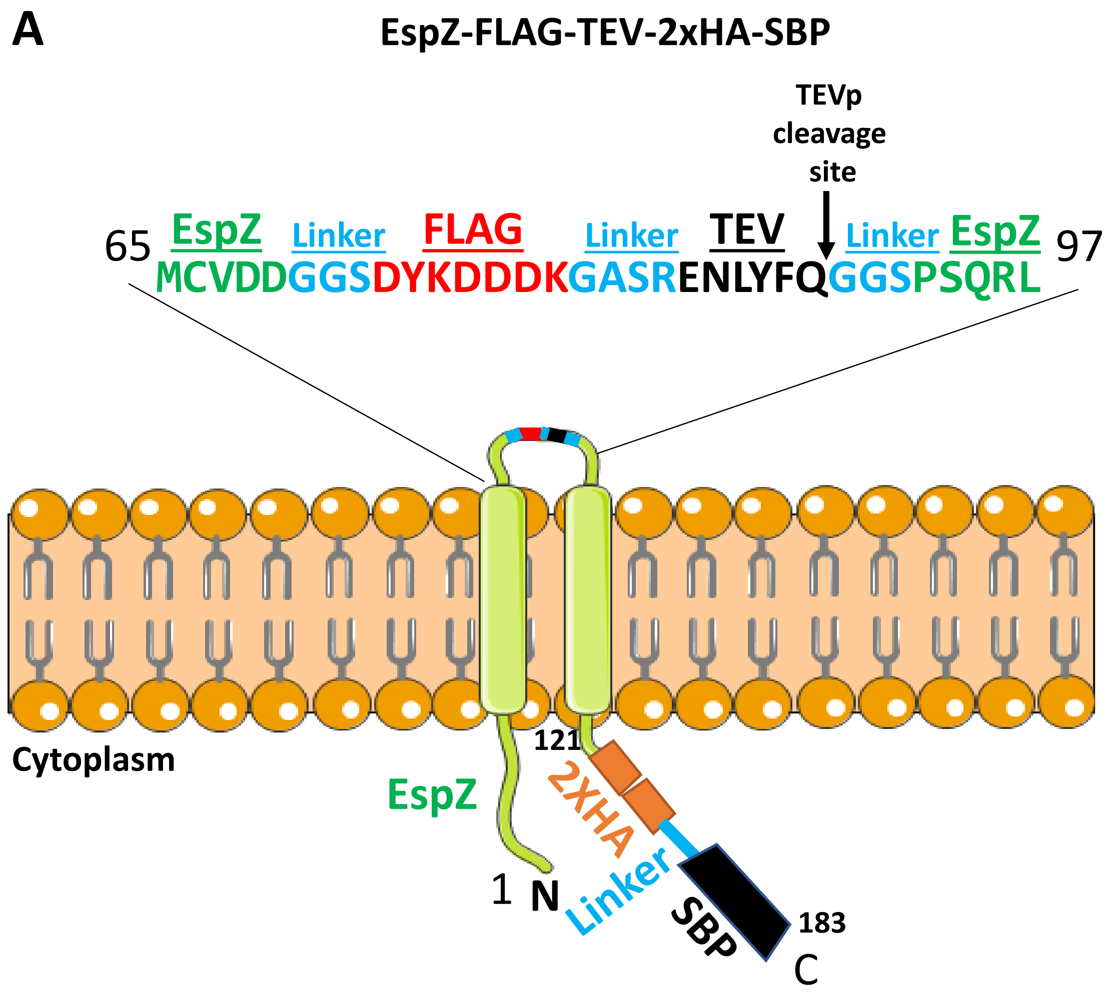
A
EspZ-FLAG-TEV-2xHA-SBP
TEVp cleavage site
65 EspZ Linker FLAG Linker TEV Linker EspZ 97
MCVDDGGSDYKDDDKGASRENLYFQGGSPSQRL
Cytoplasm
EspZ
121
2XHA
Linker
SBP
183
1 N
C

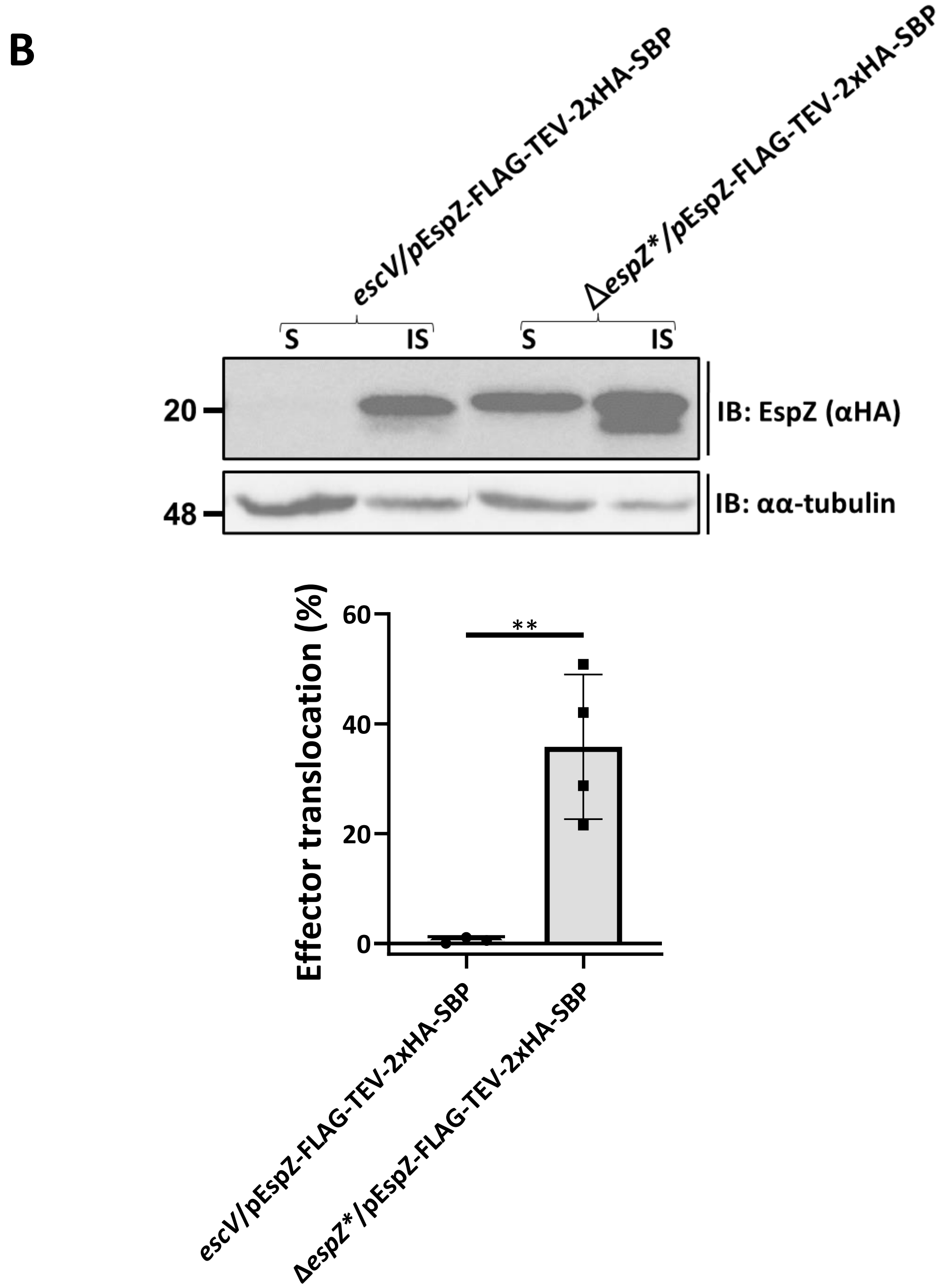
B
escV/pEspZ-FLAG-TEV-2xHA-SBP
ΔespZ*/pEspZ-FLAG-TEV-2xHA-SBP
S IS S IS
20
48
IB: EspZ (αHA)
IB: αα-tubulin
Effector translocation (%)
60
40
20
0
**
escV/pEspZ-FLAG-TEV-2xHA-SBP
ΔespZ*/pEspZ-FLAG-TEV-2xHA-SBP

Fig. S4

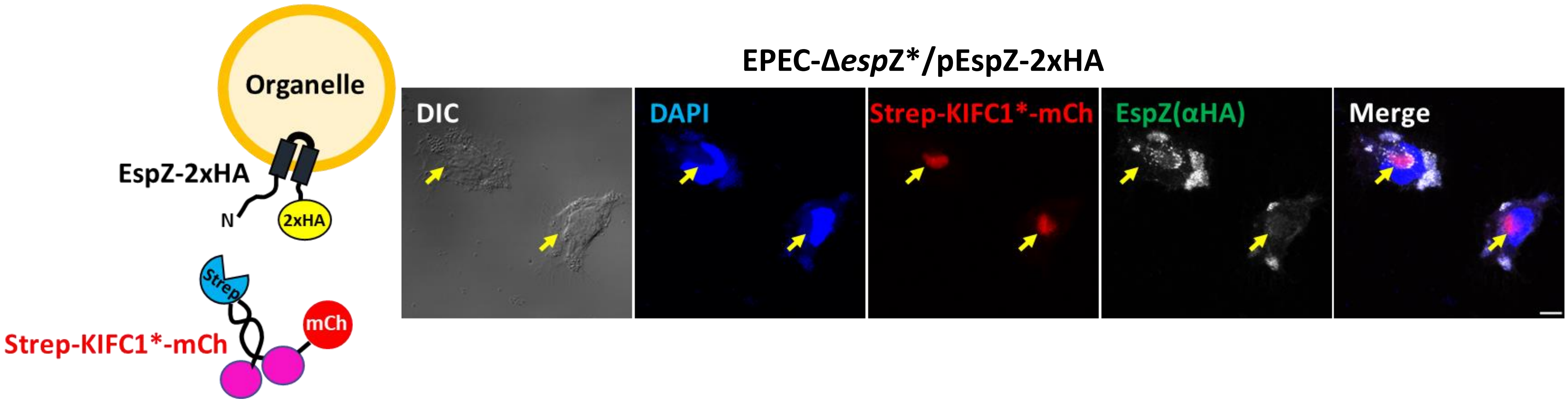
Organelle
EspZ-2xHA
N
2xHA
Strep
mCh
Strep-KIFC1*-mCh
EPEC-Δ*espZ**/pEspZ-2xHA
DIC
DAPI
Strep-KIFC1*-mCh
EspZ(αHA)
Merge

Fig. S5

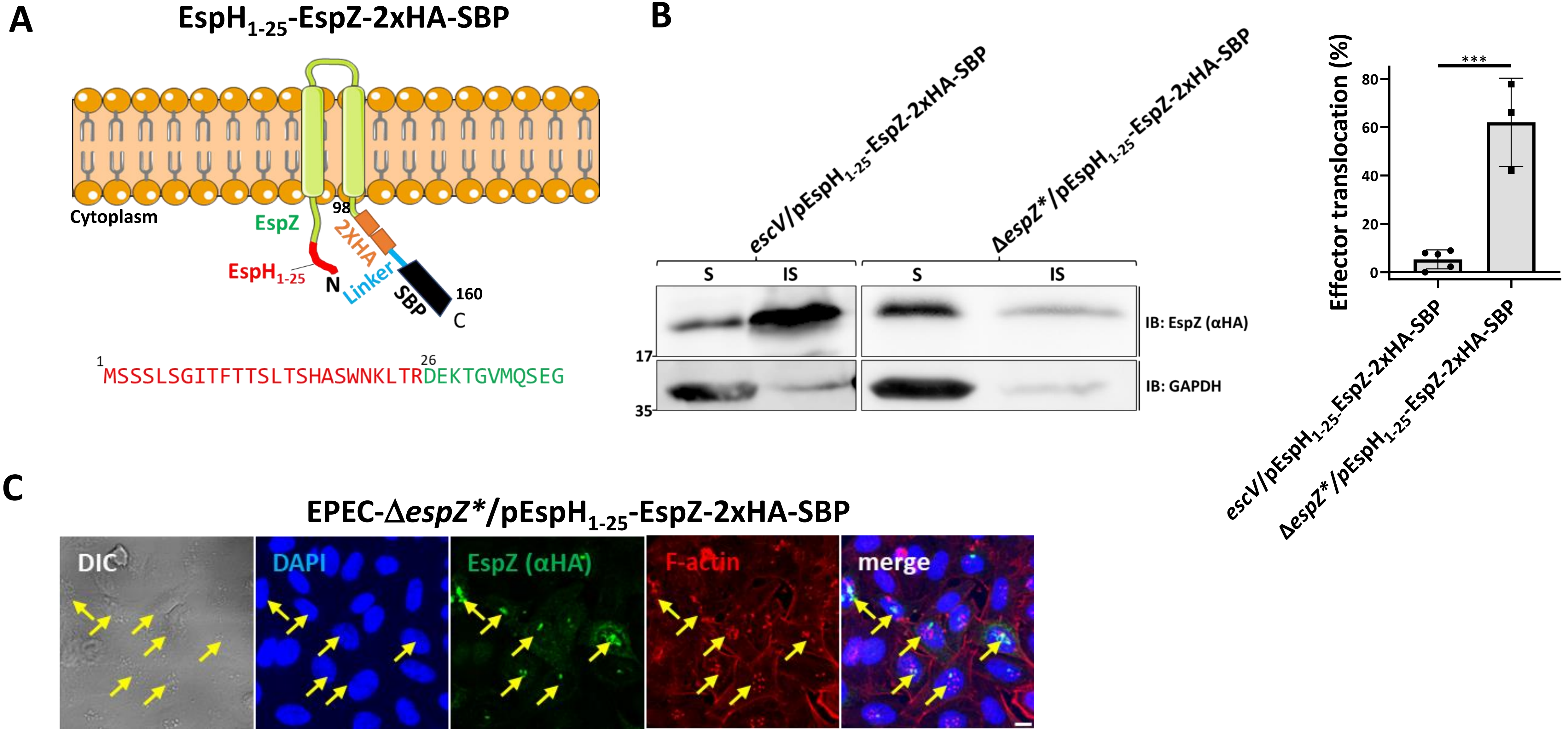
A
EspH1-25-EspZ-2xHA-SBP
Cytoplasm
EspZ
98
2XHA
EspH1-25
N
Linker
SBP
160
C
1 MSSSLSGITFTTSLTSHASWNKLTR 26 DEKTGVMQSEG
B
escV/pEspH1-25-EspZ-2xHA-SBP
ΔespZ*/pEspH1-25-EspZ-2xHA-SBP
S
IS
S
IS
IB: EspZ (αHA)
IB: GAPDH
17
35
Effector translocation (%)
***
80
60
40
20
0
escV/pEspH1-25-EspZ-2xHA-SBP
ΔespZ*/pEspH1-25-EspZ-2xHA-SBP
C
EPEC-ΔespZ*/pEspH1-25-EspZ-2xHA-SBP
DIC
DAPI
EspZ (αHA)
F-actin
merge

Fig. S6

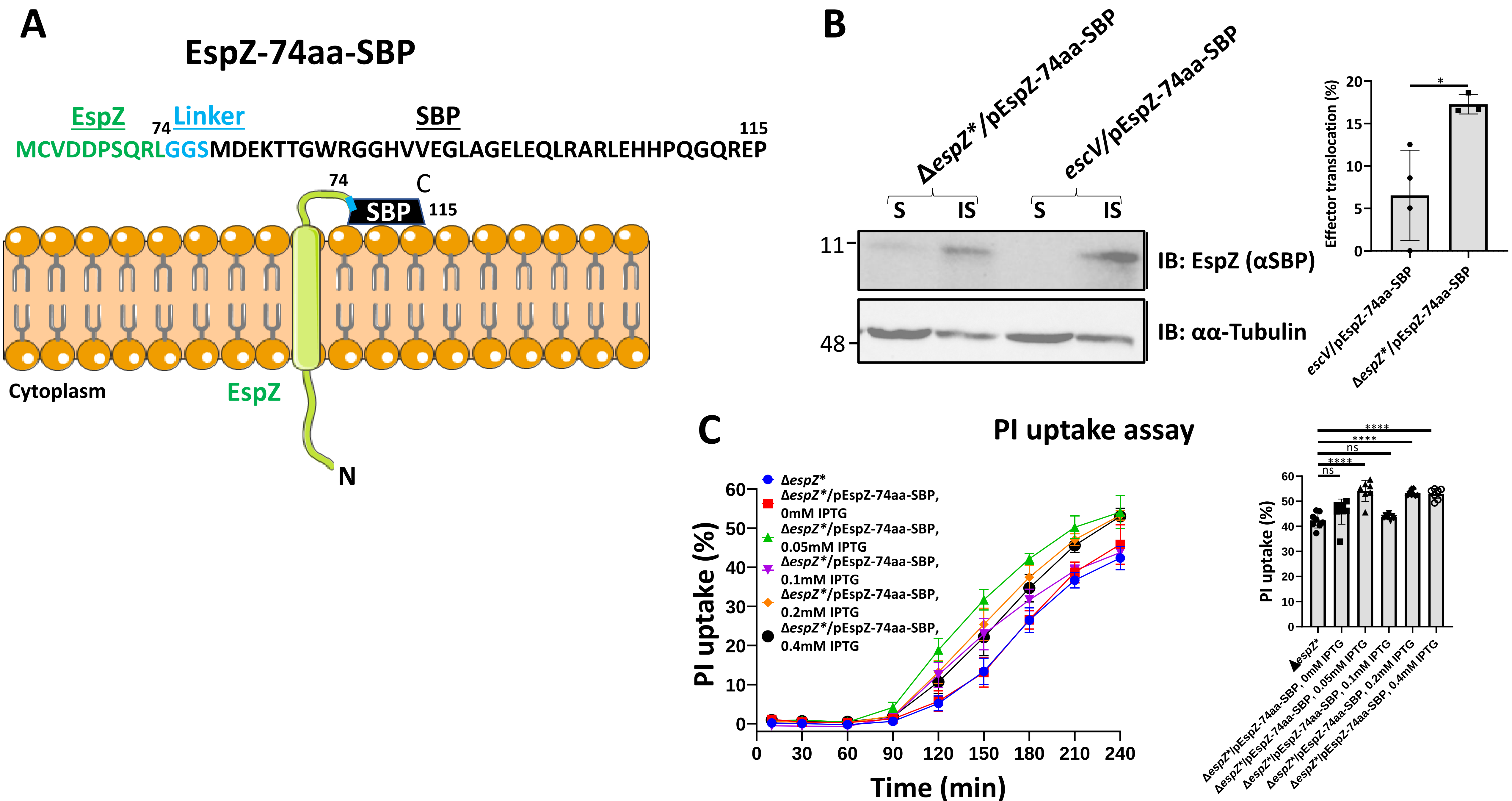

A
EspZ-74aa-SBP
EspZ
74
Linker
SBP
115
MCVDDPSQRLGGSMDEKTTGWRGGHVVEGLAGELEQLRARLEHHPQGQREP
74
C
SBP
115
Cytoplasm
EspZ
N
B
ΔespZ*/pEspZ-74aa-SBP
escV/pEspZ-74aa-SBP
S
IS
S
IS
11
48
IB: EspZ (αSBP)
IB: αα-Tubulin
Effector translocation (%)
escV/pEspZ-74aa-SBP
ΔespZ*/pEspZ-74aa-SBP
C
PI uptake assay
ΔespZ*
ΔespZ*/pEspZ-74aa-SBP, 0mM IPTG
ΔespZ*/pEspZ-74aa-SBP, 0.05mM IPTG
ΔespZ*/pEspZ-74aa-SBP, 0.1mM IPTG
ΔespZ*/pEspZ-74aa-SBP, 0.2mM IPTG
ΔespZ*/pEspZ-74aa-SBP, 0.4mM IPTG
PI uptake (%)
Time (min)
ns
****

Fig. S7

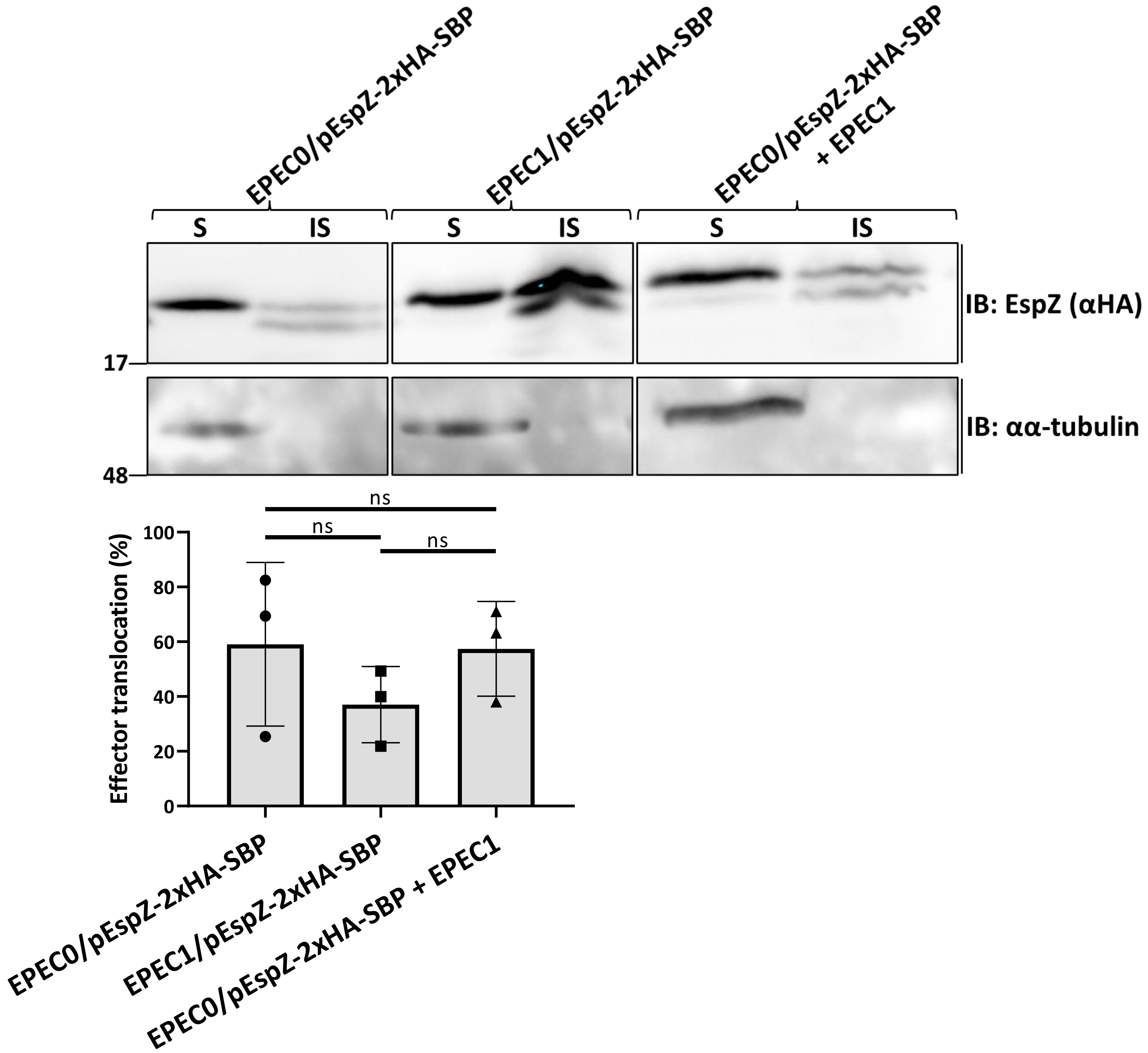

EPEC0/pEspZ-2xHA-SBP
EPEC1/pEspZ-2xHA-SBP
EPEC0/pEspZ-2xHA-SBP + EPEC1
S
IS
S
IS
S
IS
IB: EspZ (αHA)
IB: αα-tubulin
17
48
ns
ns
ns
Effector translocation (%)
100
80
60
40
20
0
EPEC0/pEspZ-2xHA-SBP
EPEC1/pEspZ-2xHA-SBP
EPEC0/pEspZ-2xHA-SBP + EPEC1

Fig. S8

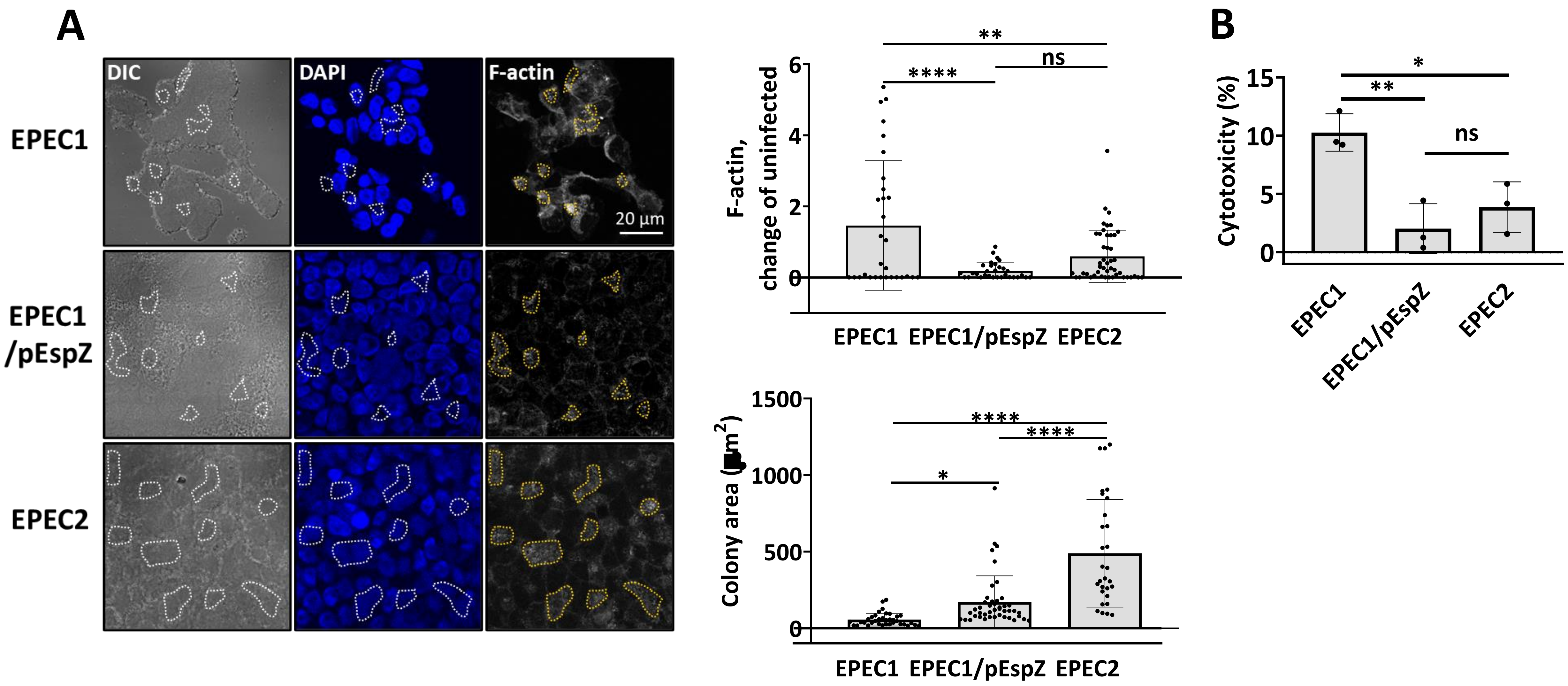
A
DIC
DAPI
F-actin
20 µm
EPEC1
EPEC1
/pEspZ
EPEC2
F-actin,
change of uninfected
6
4
2
0
**
****
ns
EPEC1
EPEC1/pEspZ
EPEC2
Colony area (µm²)
1500
1000
500
0
****
****
*
B
Cytotoxicity (%)
15
10
5
0
*
**
ns
