# Supplementary material for: Topology and function of translocated EspZ
Source: mBio. 2023 Jun 21;14(4):e00752-23. doi: 10.1128/mbio.00752-23 (PMC10470495; doi:10.1128/mbio.00752-23)
Supplement: Supplemental Tables — Tables S1 to S4. [file mbio.00752-23-s0002.pdf]

Table S1. EPEC strains:

Strain name (number)	Description	Source/Reference	Comments
E2348/69 (BA#250, IR#1)	EPEC isolate (E2348/69), serotype O127:H6, $Strep^r$	Prof. Ilan Rosenshine (HUJI)	Wild-type EPEC strain isolate
*esc*V (BA#253, IR#1961)	*escV*::*miniTn5Kn*^{r} (SN9)	Prof. Ilan Rosenshine (HUJI)(1)	T3SS deficient EPEC mutant
Δ*espZ** (BA#1143, IR#5566)	Δ*espZ*, Δ*pp4* (*nle*G, *nle*B2, *nle*C, *nle*H fragment, *nle*D), ΔIE6 (espL, *nle*B1, *nle*E1, *efa1/lifA*)::Kn^r	Prof. Ilan Rosenshine (HUJI)	An *espZ, pp4 and IE6* deficient EPEC mutant
ΔespZ/*pEspZ-2xHA-SBP (BA#1224)	Δ*espZ** strain complemented with pSA10-EspZ-2XHA-SBP plasmid:: Amp^r, Kn^r	Present study	An *espZ* EPEC mutant complemented with an EspZ-2xHA-SBP encoding plamsid
*esc*V/pEspZ-2xHA-SBP (BA#1251)	Δ*escV* strain complemented with pSA10-EspZ-2XHA-SBP plasmid:: Amp^r, Kn^r	Present study	An EPEC mutant lacking the *escV* gene, and complemented with an EspZ-2xHA-SBP encoding plasmid
Δ*espZ**/pEspZ-loop-Flag-TEV-2xHA-SBP (BA#1240)	Δ*espZ** strain complemented with pSA10-EspZ-loop-Flag-TEV-2xHA-SBP plasmid:: Amp^r, Kn^r	Present study	An *espZ* EPEC mutant complemented with an EspZ-loop-Flag-TEV-2xHA-SBP encoding plasmid
*esc*V/pEspZ-loop-Flag-TEV-2xHA-SBP (BA#1226)	*esc*V strain complemented with pSA10-EspZ-loop-Flag-TEV-2xHA-SBP plasmid:: Amp^r, Kn^r	Present study	An EPEC mutant lacking the *escV* gene, and complemented with an EspZ-loop-Flag-TEV-2xHA-SBP encoding plasmid
Δ*espZ**/pEspH_{1-25}-EspZ-2xHA-SBP (BA#1274)	Δ*espZ** strain complemented with pSA10-EspH_{1-25}-EspZ-2xHA-SBP plasmid:: Amp^r, Kn^r	Present study	An *espZ* EPEC mutant complemented with an EspH1-25-EspZ- 2xHA-

			SBP encoding plasmid
*esc*V/pEspH$_{1-25}$-EspZ-2xHA-SBP (BA#1227) (BA#1274)	*escV* strain complemented with pSA10- EspH$_{1-25}$-EspZ-2xHA-SBP plasmid:: Amp^r, Kn^r	Present study	An EPEC mutant lacking the *escV* gene, and complemented with an EspH$_{1-25}$-EspZ-2xHA-SBP encoding plasmid
Δ*espZ**/pEspZ-74aa-SBP (BA#1241)	Δ*espZ** strain complemented with pSA10-EspZ-74aa-SBP plasmid:: Amp^r, Kn^r	Present study	An *espZ* EPEC mutant complemented with an EspZ-74aa-SBP encoding plasmid
*escV/*pEspZ-74aa-SBP (BA#1288)	*esc*V strain complemented with pSA10-EspZ-74aa-SBP plasmid:: Amp^r, Kn^r	Present study	An EPEC mutant lacking the *escV* gene, and complemented with an EspZ-74aa-SBP encoding plasmid
EPEC0 (BA#1269)	E2348/69 lacking any effector encoding genes::Amp, Strep^r	(2)	EPEC which does not express type III secreted effectors
EPEC1 (BA#1274)	E2348/69 containing the *tir* encoding gene Amp, Strep^r	(2)	EPEC which expresses Tir only
EPEC2 (BA#1274)	E2348/69 containing *tir* and *esp*Z encoding genes Amp, Strep^r	(2)	EPEC which expresses Tir and EspZ only
Δ*espZ**/pEspZ-2XHA (BA#1225)	Δ*espZ** strain complemented with pSA10-EspZ-2XHA plasmid::Amp^r, Kn^r	Present study	An *espZ* EPEC mutant complemented with an EspZ-2XHA encoding plasmid
esc*V/*pEspZ-2XHA (BA#1286)	*escV* strain complemented with pSA10-EspZ-2XHA plasmid::Amp^r, Kn^r	Present study	An EPEC mutant lacking the *escV* gene, and complemented with an EspZ-

			2xHA encoding plasmid

Footnote: All mutant strains are derivatives of E2348/69. The initials BA (Benjamin Aroeti) and IR (Ilan Rosenshine) refer to the investigators in whose lab the strains have been made.

References

1. Nadler C, Shifrin Y, Nov S, Kobi S, Rosenshine I. 2006. Characterization of enteropathogenic Escherichia coli mutants that fail to disrupt host cell spreading and attachment to substratum. Infect Immun 74:839-49.
2. Cepeda-Molero M, Berger CN, Walsham ADS, Ellis SJ, Wemyss-Holden S, Schuller S, Frankel G, Fernandez LA. 2017. Attaching and effacing (A/E) lesion formation by enteropathogenic E. coli on human intestinal mucosa is dependent on non-LEE effectors. PLoS Pathog 13:e1006706.

Table S2: Primary and secondary antibodies

Antibody	Primary/Secondary dilution (IF/WB)	Description
Mouse anti-SBP	Primary<br>1:400 (IF)<br>1:2000 (WB)	Streptavidin Binding Protein (SBP) tag antibody (SB19-C4); sc101595; Santa Cruz Biotechnology
Rabbit anti-HA	Primary<br>1:400 (IF)<br>1:2000 (WB)	Rabbit monoclonal anti-HA tag antibody (C29F4); Cell Signaling
Mouse anti-FLAG tag	Primary<br>1:400 (IF)	Mouse monoclonal ANTI-FLAG® M2 antibody (F-1804); Sigma-Aldrich
Mouse anti-α-tubulin	Primary<br>1:2000 (WB)	Mouse monoclonal Anti-α-tubulin antibody (T6074); Sigma Aldrich
Mouse anti-GAPDH	Primary<br>1:200 (WB)	Mouse monoclonal anti GAPDH antibody; sc-47724 Santa Cruz biotechnology inc.
Goat anti-mouse IgG, Alexa Fluor 488	Secondary<br>1:300 (IF)	Alexa Fluor 488 - goat anti-mouse IgG; (115-545-062) Invitrogen; Life Technologies/ThermoFisher
Donkey anti-mouse IgG, Alexa Fluor 594	Secondary<br>1:300 (IF)	Alexa Fluor 594 - donkey anti-mouse IgG; (A-21203) Invitrogen; Life Technologies/ThermoFisher

Goat anti-Rabbit IgG, Alexa Fluor 488	Secondary 1:300 (IF)	Alexa Fluor 488 - goat anti-rabbit IgG; (A11008); Invitrogen; Life Technologies/ThermoFisher
Goat anti-mouse IgG, Alexa Fluor 594	Secondary 1:300 (IF)	Alexa Fluor 594 - goat anti-rabbit IgG; (A-11029) Invitrogen; Life Technologies/ThermoFisher
Donkey anti-mouse Cy5 (Alexa Fluor 647)	Secondary 1:300 (IF)	Cy5 - donkey anti-mouse IgG; (715-175-151) Jackson ImmunoResearch Laboratories
Donkey anti-rabbit Cy5 (Alexa Fluor 647)	Secondary 1:300 (IF)	Cy5 - donkey anti-rabbit IgG; (711-602-152) Jackson ImmunoResearch Laboratories
Peroxidase goat anti-mouse IgG	Secondary 1:10000 (WB)	Peroxidase- AffiniPure Goat Anti-Mouse IgG; (115-035-166); Jackson ImmunoResearch Laboratories
Peroxidase goat anti-rabbit IgG	Secondary 1:10000 (WB)	Peroxidase- AffiniPure Goat Anti-Rabbit IgG; 111-035-003; Jackson ImmunoResearch Laboratories

Table S3: Plasmids

**Plasmid name**	**Description**	**Reference**
pSA10	Bacterial expression IPTG inducible vector, Amp^r	(1)
pSA10-EspH-6xHIS-SBP (pAA6284)	pSA10 encoding EspH tagged with 6xHIS and SBP at the C-terminus	(2)
pEspZ-mCherry	A mammalian expression vector that expresses mCherry fused to the C-terminus of EspZ	Prof. Ilan Rosenshine (HUJI)
pSA10-EspZ-2xHA-SBP	pSA10 encoding EspZ tagged with a GGSGGS linker, tandem HA tag, and SBP tag at the C-terminus	This study; **Fig. S1**
pSA10-EspZ-2xHA	pSA10 encoding EspZ with a GGSGGS linker and tandem 2xHA tags at the C-terminus	This study; **Fig. S2A**
pSA10-EspZ-FLAG-TEV-2xHA-SBP	pSA10 encoding EspZ-2xHA-SBP containing FLAG and TEV protease cleavage tags introduced into the putative extracellular loop of EspZ	This study; **Fig. S3A**
pSA10-$EspH_{1-25}$-EspZ-2xHA-SBP	pSA10 encoding EspZ-2xHA-SBP in which aa 1-25 of EspZ have been swapped with aa 1-25 of EspH	This study; **Fig. S4A**
pSA10-EspZ-74aa-SBP	pSA10 encoding EspZ in which aa 75-119 were removed in-frame from the pSA10-EspZ-2xHA-SBP encoding plasmid	This study; **Fig. S5A**
pcDNA3.1-EspZ-2xHA-SBP	A pcDNA3.1 mammalian expression vector that expresses EspZ-2xHA-SBP	This study
pcDNA3.1-EspZ-FLAG-TEV-2xHA-SBP	A pcDNA3.1 mammalian expression vector that expresses EspZ-FLAG-TEV-2xHA	This study

pcDNA3.1-EspH$_{1-25}$-EspZ-2xHA-SBP	A pcDNA3.1 mammalian expression vector that expresses EspH$_{1-25}$-EspZ-2xHA-SBP	This study
pcDNA3.1-EspZ-74aa-SBP	A pcDNA3.1 mammalian expression vector that expresses EspZ-74aa-SBP	This study
4xMTS-mNeonGreen	A mammalian expression vector encoding a mNeonGreen tagged mitochondrial marker	Addgene #98876
Strep-KIFC1*-mCherry	A mammalian expression vector that expresses a Streptavidin and mCherry tagged KIFC1*	Juan Bonifacino (NIH) (3)
pcDNA3.1-GFP (1-10)	A pcDNA3.1 mammalian expression vector that expresses GFP(1-10)	Addgene #70219

References

1. Schlosser-Silverman E, Elgrably-Weiss M, Rosenshine I, Kohen R, & Altuvia S (2000) Characterization of Escherichia coli DNA lesions generated within J774 macrophages. *J Bacteriol* 182(18):5225-5230.
2. Ramachandran RP*, et al.* (2018) EspH Suppresses Erk by Spatial Segregation from CD81 Tetraspanin Microdomains. *Infection and immunity* 86(10):00303-00318.
3. Guardia CM*, et al.* (2019) Reversible association with motor proteins (RAMP): A streptavidin-based method to manipulate organelle positioning. *PLoS Biol* 17(5):e3000279.

Table S4. List of primers and their usage

S. No.	Name	Sequence	Usage
1F'	pSA10-SBP F' linear GA	ATGGACGAAAAAACCACCGGTT	Generation of pSA10-EspZ-2xHA-SBP
2R'	pSA10-EspZ tail R' linear GA	CTTAAATTTGCTGCTTCCATAATTCTGTTTCCTGTGTGAAATTGTTATCCG	Generation of pSA10-EspZ-2xHA-SBP
3F'	EspZ F' GA	ATGGAAGCAGCAAATTTAAGCCCTTC	Generation of pSA10-EspZ-2xHA-SBP and pSA10-EspZ-2xHA
4R'	EspZ R' GA	GGCATATTTCATCGCTAATCCGCCG	Generation of pSA10-EspZ-2xHA-SBP and pSA10-EspZ-2xHA
5F'	2xHA EspZ tail F' GA	GATTAGCGATGAAATATGCCTATCCATACGATGTGCCTGATTATGC	Generation of pSA10-EspZ-2xHA-SBP and pSA10-EspZ-2xHA
6R'	2xHA SBP tail R' GA	CCGGTGGTTTTTTCGTCCATACTTCCACCGCTTCCCCC	Generation of pSA10-EspZ-2xHA-SBP
7	Tags	TATCGGTACTGGTATCGCAGCAATGGGAGGTTCTGGCGGGAGCTATCCATACGATGTGCCTGATTATGCGTACCCCTATGATGTGCCGGATTACGCGGGGGGAAGCGGTGGAAGTTGGAGTCATCCGAATTTCCAAAAGGGTGGCAGCGGTGGAAGCTGGAGCCACCCAAACTTTCAGAAAGGTGGCAGTGGAGGCAGCTTAGGCTTAGGAATCGCAGCCGGTG	Generation of pSA10-EspZ-2xHA-SBP and pSA10-EspZ-2xHA
8F'	pSA10 linear F'	ACAATTCGCGCGCGAAGGC	Generation of pSA10-EspZ-2xHA
9R'	2xHA pSA tail R' GA	GCCTTCGCGCGCGAATTGTTTACGCGTAATCCGGCACATCAT	Generation of pSA10-EspZ-2xHA
10F'	Linear 1xFLAG-1xTEV F'	GATAAAGGCGCCTCTAGAGAAAACCTGTATTTTCAGGGCGGCAGCCCTTCACAAAGATTAGGCTT	Generation of pSA10-EspZ-FLAG-TEV-2xHA-SBP
11R'	Linear 1xFLAG-1xTEV R'	GTTTTCTCTAGAGGCGCCTTTATCATCATCTTTATAATCGCTGCCGCCGTCGTCAACACACATTGCTG	Generation of pSA10-EspZ-FLAG-TEV-2xHA-SBP
12F'	espZ 26 F'	GATGAGAAGACAGGGGTGATGCAGT	Generation of pSA10-EspH$_{1-25}$-EspZ-2xHA-SBP
13R'	pSA10 R'	GAATTCTGTTTCCTGTGTGAAATTGTTATCCGC	Generation of pSA10-EspH$_{1-25}$-EspZ-2xHA-SBP
14F'	espH 25 F'	CAATTTCACACAGGAAACAGAATTCATGAGC	Generation of pSA10-EspH$_{1-25}$-EspZ-2xHA-SBP

15R'	espH 25 R'	ACTGCATCACCCCTGTCTTCTCATCGCGGGTCAGTTTGTTCCAGCT	Generation of pSA10-EspH$_{1-25}$-EspZ-2xHA-SBP
16	gBlock 25aa EspH	CAATTTCACACAGGAAACAGAATTCATGAGCAGCAGCCTGAGCGGCATTACCTTTACCACCAGCCTGACCAGCCATGCGAGCTGGAACAAACTGACCCGCTCGAACCTTATGATTAATCATGGCAAAC	Generation of pSA10-EspH$_{1-25}$-EspZ-2xHA-SBP
17F'	pcDNA linear F'	TCTAGAGGGCCCGTTTAAACCC	Generation of pcDNA3.1-EspZ-2xHA-SBP and pcDNA3.1-EspZ-FLAG-TEV-2xHA-SBP
18R'	pcDNA linear R'	AGATCTGTTAACGAATTCCACCACA	Generation of pcDNA3.1-EspZ-2xHA-SBP and pcDNA3.1-EspZ-FLAG-TEV-2xHA-SBP
19F'	1240 pcDNA tail F'	TGGAATTCGTAAACAGATCTATGGAAGCAGCAAATTTAAGCCCTTC	Generation of pcDNA3.1-EspZ-2xHA-SBP and pcDNA3.1-EspZ-FLAG-TEV-2xHA-SBP
20R'	1240 pcDNA tail R'	GTTTAAACGGGCCCTCTAGATTACGGTTCACGCTGACCCTGC	Generation of pcDNA3.1-EspZ-2xHA-SBP and pcDNA3.1-EspZ-FLAG-TEV-2xHA-SBP
21R'	pcDNA espH tail R'	GAATTCTGTTTCCTGTGTGAAATTGCTGGACTAGTGGATCCGAGCTCG	Generation of pcDNA3.1-EspH$_{1-25}$-EspZ-2xHA-SBP
22R'	linear-espz-74aa-sbp tail R	CCGGTGGTTTTTTCGTCCATACTTCCACCTAATCTTTGTGAAGGGTCGT	Generation of pSA10-EspZ-74aa-2xHA-SBP
23F'	1240 pcDNA tail F	TGGAATTCGTTAACAGATCTATGGAAGCAGCAAATTTAAGCCCTT	Generation of pcDNA3.1-EspZ-74aa-2xHA-SBP
24R'	1240 pcDNA tail R	GTTTAAACGGGCCCTCTAGATTACGGTTCACGCTGACCCTGCGGGTGG	Generation of pcDNA3.1-EspZ-74aa-2xHA-SBP
