# Supplementary material for: Topology and function of translocated EspZ
Source: mBio. 2023 Jun 21;14(4):e00752-23. doi: 10.1128/mbio.00752-23 (PMC10470495; doi:10.1128/mbio.00752-23)
Supplement: Supplemental Legends — Legends to Fig. S1 to S8. [file mbio.00752-23-s0003.docx]

**Supplementary figure legends:**

**Fig. S1**: ***Schematic of EspZ-2xHA-SBP***. The 98 aa EspZ of EPEC O127:H6 (strain E2348/69) is illustrated in green and the context of a membrane lipid bilayer. The effector has been predicted to contain an extracellular loop (residues 65-74), two transmembrane domains, and N and C-termini facing the host cell cytoplasm ([14](#_ENREF_14)). See also: <https://www.uniprot.org/uniprotkb/B7UMA9/entry>). Two tandem influenza hemagglutinin (HA) peptide tags (orange), followed by a GGSGGS linker (blue) and a subsequent 38aa streptavidin binding protein (SBP; black) were introduced after the C-terminal alanine (A98) residue of EspZ. The amino acid sequence of the C-terminal portion of the EspZ construct is shown in a single-letter code.

**Fig. S2: *Characterization of EspZ-2xHA*. (A) Schematic description of the effector.** The effector is the same as in **Fig. S1** but bears only two tandem HA tags in its C-terminus. The amino acid sequence of its C-terminus is indicated in a single-letter code. **(B) Immunofluorescence analysis.** HeLa cells were infected with EPEC-ΔespZ*/pEspZ-2xHA for 60 min at 37 °C, fixed, and processed for immunofluorescence microscopy using anti-HA antibodies, as described in Materials and Methods. A representative image out of three independent experiments is shown. Scale bar, 10 µm. **(C)** **EspZ-2xHA translocation.** The effector translocation assay was applied, as described in Materials and Methods. A representative IB (out of 3 independent experiments) (**upper)** and the calculated effector translocation levels in percentage (**bottom)** are shown. ** p-value≤0.01. **(D) Effects on lytic cell death.** HeLa cells were infected with the indicated bacterial strains, and under different IPTG concentrations, and the LDH release or PI uptake assays were performed, as described in Materials and Methods and **Fig.1C**. Results are mean ± SD of 3 or 4 independent measurements. **** P <0.0001, *** p-value<0.001, non-significant (ns) p-value>0.05.

**Fig. S3:** ***EspZ-FLAG-TEV-2xHA-SBP is translocated into the infected HeLa cells***. (**A) Schematic description of the mutant effector.** The effector is identical to that described in **Fig. S1**, except that FLAG (red) and TEV (black) tag sequences have been introduced into the putative extracellular loop of the effector (green). The amino acid sequence is presented in a single-letter code and the TEVp cleavage site is pointed with an arrow. **(B)** **Effector translocation**. The effector translocation assay was performed, as described in Materials and Methods. A representative gel out of 4 independent experiments (upper) and quantitative translocation levels (bottom) are shown. Results are presented as the mean ± SD. ** p-value≤0.01.

**Fig. S4: *The C-terminus of translocated EspZ-2xHA does not colocalize with Strep-KIFC1*-mCh.*** This control experiment was done as described in **Fig. 2B**, except that the Strep-KIFC1*-mCh expressing cells were infected with EPEC-Δ*espZ**/pEspZ-2xHA. The absence of C-terminal SBP did not allow the interactions between the Streptavidin of Strep-KIFC1*-mCh and the effector, resulting in a lack of colocalization between the two proteins (see illustration). Arrows point towards infecting bacterial microcolonies. Representative images (from 3 independent experiments) are shown. Bar, 10 µm.

**Fig. S5: *Characterization of*** ***EspH_1-25_-EspZ-2xHA-SBP***. **(A) Schematic illustration of the mutant effector.** The construct is the same as in **Fig. S1**, except that the N-terminal 25aa sequence of EspZ has been replaced with the N-terminal 25aa sequence of EspH (red). A single code letter is used to show the amino acid sequence of the EspZ (green)-EspH (red) interface. **(B) Effector translocation.** The translocation assay was performed, as described in Materials and Methods. A representative gel (out of 3 or 4 experiments) (**left**) and quantitative analyses (**right**) are shown. Results are presented as the mean ± SD. *** p-value≤0.001. **(C) Immunofluorescence analysis*.*** HeLa cells were infected with EPEC-Δ*espZ**/pEspH_1-25_-EspZ-2xHA-SBP for 60 min at 37 °C, and the cells were immunostained with anti-HA antibodies (to label EspZ), and stained with DAPI (DNA), and TR-phalloidin (F-actin). Cells were then processed for confocal imaging, and representative images (out of 3 independent experiments) are shown. Arrows point toward infecting bacterial microcolonies. Bar, 10 µm.

**Fig. S6: Characterization of EspZ-74aa-SBP. (A) Schematic illustration of the mutant effector.** A GGS linker (blue) followed by SBP (black) has been introduced immediately after L74, which is the last amino acid of the predicted extracellular loop of EspZ (green), generating the EspZ-74aa-SBP mutant. The amino acid sequence of the EspZ-Linker-SBP (black) is shown in a single-letter code. **(B) Effector translocation.** The translocation assay was performed, as described in Materials and Methods. A representative image of the gel (out of 3 - 4 experiments) (left) and the quantitative analysis (right) are shown. Results are presented as the mean ± SD. * p-value≤0.05. **(C) Effects on lytic cell death.** HeLa cells were infected with the indicated bacterial strains, and under different IPTG concentrations, and the PI uptake assay was performed, as described in Materials and Methods and **Fig.1C**. Results are mean ± SD of 6 independent measurements. **** P <0.0001, non-significant (ns) p-value>0.05.

**Fig. S7: EspZ translocation into EPEC0/pEspZ-2xHA-SBP, EPEC1/pEspZ-2xHA-SBP and EPEC0/pESpZ-2xHA-SBP+EPEC1 infected cells.** HeLa cells were infected with the indicated and primed EPEC strains for 120 min at 37 °C. Cells were then subjected to the ‘effector translocation assay’ described in Materials and Methods. A representative gel (out of 3 independent experiments) (upper) and quantitative analysis (bottom) are shown. The results show that the effector was translocated at roughly equivalent levels to those displayed by EspZ-2xHA-SBP (**Fig. 1B**). Results are presented as the mean ± SD. non-significant (ns) p-value>0.05.

**Fig. S8: *EspZ effects on Tir-induced F-actin pedestals, microcolony area, and lytic cell death of Caco-2_BBe_ cells.* (A) Effects on pedestals and microcolony area.** CaCo-2_BBe_ cells were seeded on a collagenated 24-well plate and grown for 18-21 days before the experiment, as described in Material and Methods. Cells were infected with primed EPEC strains for 180 min at 37 °C, and then fixed, permeabilized, and stained with DAPI (DNA), and TR-phalloidin (F-actin) and imaged by confocal microscopy. Representative images (out of 3 experiments) are shown (**left**). Bar, 20 µm. Cell-attached bacterial microcolonies are marked with a dotted line. The TR-phalloidin fluorescence intensity levels at infection sites, which represent F-actin-rich pedestals, were measured by Fuji (NIH) and normalized to fluorescence background levels. Results are mean ± SD measured for ~20 microcolonies (**upper right**). The area of marked microcolonies was also determined by Fiji (NIH) and shown as a bar graph (**bottom right**). Results are mean ± SD measured for ~20 microcolonies. **** p-value<0.0001; ** p-value<0.002; ns; non-significant p-value>0.05. (**B) Effects on lytic cell death measured by LDH release assay.** As before, cells were infected with primed EPEC strains, and the LDH release assay was performed, as described in Materials and Methods. Results are mean ± SD of 3-6 measurements. **** p-value<0.0001, non-significant (ns) p-value>0.05.
